# Transcriptome profiling and comparison of maize ear heterosis during the spikelet and floret differentiation stages

**DOI:** 10.1186/s12864-016-3296-8

**Published:** 2016-11-22

**Authors:** Xiaojiao Hu, Hongwu Wang, Xizhou Diao, Zhifang Liu, Kun Li, Yujin Wu, Qianjin Liang, Hui Wang, Changling Huang

**Affiliations:** National Key Facility for Crop Gene Resources and Genetic Improvement, Institute of Crop Science, Chinese Academy of Agricultural Sciences, No.12 Zhongguancun South Main Street, Beijing, 100081 China

**Keywords:** Maize (*Zea mays L.*), Ear development, Heterosis, Transcriptomics, Additive gene expression, Allele-specific expression, *cis*- and *trans*-regulation

## Abstract

**Background:**

Hybridization is a prominent process in the evolution of crop plants that can give rise to gene expression variation, phenotypic novelty and heterosis. Maize is the most successful crop in utilizing heterosis. The development of hybrid maize ears exhibits strong heterotic vigor and greatly affects maize yield. However, a comprehensive perspective on transcriptional variation and its correlation with heterosis during maize ear development is not available.

**Results:**

Using RNA sequencing technology, we investigated the transcriptome profiles of maize ears in the spikelet and floret differentiation stages of hybrid ZD808 and its parents CL11 and NG5. Our results revealed that 53.9% (21,258) of maize protein-coding genes were transcribed in at least one genotype. In both development stages, significant numbers of genes were differentially expressed between the hybrid and its parents. Gene expression inheritance analysis revealed approximately 80% of genes were expressed additively, which suggested that the complementary effect may play a foundation role in maize ear heterosis. Among non-additively expressed genes, NG5-dominant genes were predominant. Analyses of the allele-specific gene expression in hybrid identified pervasive allelic imbalance and significant preferential expression of NG5 alleles in both developmental stages. The results implied that NG5 may provide beneficial alleles that contribute greatly to heterosis. Further comparison of parental and hybrid allele-specific expression suggested that gene expression variation is largely attributable to *cis*-regulatory variation in maize. The *cis*-regulatory variations tend to preserve the allelic expression levels in hybrid and result in additive expression. Comparison between the two development stages revealed that allele-specific expression and *cis-*/*trans*-regulatory variations responded differently to developmental cues, which may lead to stage-specific vigor phenotype during maize ear development.

**Conclusion:**

Our research suggests that *cis*-regulated additive expression may fine-tune gene expression level into an optimal status and play a foundation role in maize ear heterosis. Our work provides a comprehensive insight into transcriptional variation and its correlation with heterosis during maize ear development. The knowledge gained from this study presents novel opportunity to improve our maize varieties.

**Electronic supplementary material:**

The online version of this article (doi:10.1186/s12864-016-3296-8) contains supplementary material, which is available to authorized users.

## Background

Heterosis refers to the superior performance in biomass, yield, or other agronomic traits of hybrids relative to their parents [1, 2]. This phenomenon has revolutionized crop breeding and production by increasing yields from 15 to 50% [3, 4]. However, the genetic and molecular bases of heterosis are controversial. Various models have been posited to explain heterosis, including classic dominance, over-dominance and epistasis models, which have been debated for over 100 years [5–9]. However, none of the hypotheses can fully explain this important scientific phenomenon. With the development of omics technologies, studies in hybrids using genomic, transcriptomic, epigenomic and proteomic approaches have provided a new perspective into the molecular mechanisms of heterosis [1, 10, 11].

Recent researches have shown that variations in gene expression and regulatory networks are important sources of phenotypic novelty and are associated with heterosis. Comparative gene expression profiling between hybrids and their parents has been conducted in various organisms, including *Arabidopsis* [12, 13], maize [14–16], rice [17, 18] and other species [19]. Multiple modes of gene action, including additive, high- and low-parent dominance and over- and under-dominance were suggested to contribute to heterosis [15]. Several studies in maize have revealed that additive gene expression was prevalent and positively correlated with heterosis and high yield [14–16, 20]. However, in other studies, dominant or transgressive (over- and under-dominant) gene expression were suggested to be important in conferring superiority hybrid traits [21–24].

Hybridization gives rise to a vast reservoir of allelic variation that has been suggested to affect gene expression levels [2, 25, 26]. Early studies indicated that up to 50% of differentially expressed genes are affected by allele variations [27]. The interactions of two parent alleles in the hybrid are considered to be important determinants of a superior phenotype, and they can be regulated by *cis*- or *trans*-acting factors [28, 29]. *Cis*-regulation can occur as a result of variation in DNA sequences or epigenetic modifications of *cis*-regulatory elements of the nearby gene. Alternatively, *trans*-regulation is due to variation in remote *trans*-acting factors, which affect downstream gene expression levels. *Cis*-regulation changes affect gene expression in an allele-specific manner, whereas *trans*-regulation affects both alleles in the hybrid. The relevant contributions of *cis* and *trans* effects to the divergence of gene expression have been discussed in previous studies. In maize, *cis*-regulatory variation has been found to contribute greatly to parental expression divergence and is correlated with additive expression patterns in the hybrid [30, 31]. Allele-specific expression studies in maize hybrid seedlings revealed that *cis*-regulatory variation accounts for 70% of the differentially expressed genes [27]. In certain other species, *trans*-regulation has been suggested to play important roles in gene expression variation [32, 33]. However, recent evidence indicates that gene expression stability is also maintained by the coordination of *cis*- and *trans*-regulatory activity [34, 35]. These discrepancies suggests that different gene expression patterns and regulatory mechanisms may not be solely responsible for heterosis and more likely associated with particular species, tissues and developmental stages.

Maize is the most widely grown and highest-yielding crop worldwide. Immature maize ear development exhibits strong heterosis in ear architectural traits and greatly affects maize yield [36]. Ear inflorescence differentiation is a continuous, dynamic process that includes growth cone elongation, spikelet differentiation, floret differentiation and organ formation. The spikelet and floret differentiation stages are crucially important because the axillary spikelet pair meristems and floral meristems are formed during these stages, and these steps determine the two main components of maize yield, namely, kernel row number and kernel number per row [37, 38]. Study of the molecular basis of ear heterosis during these two stages could have great impact on high-yield maize breeding.

ZD808 is an excellent maize hybrid, which was bred by our research group and approved by the National Crop Variety Approval Committee of China in 2006. This variety has been recommended by the Ministry of Agriculture as leading variety for southwestern China for eight consecutive years. ZD808 exhibits strong heterosis in ear architectural traits, with large ears, large grains and high grain yield (Table 1), and it is an excellent model for the molecular investigation of ear heterosis. ZD808 was derived from a cross between the inbred lines CL11 and NG5. The maternal line CL11 has tropical genetic components and exhibits a high resistance to stress and disease, whereas the paternal line NG5 has large ears and a high, stable yield.

**Table 1:** Heterosis analysis of ear architectural traits of ZD808

Stage	Trait	NG5^a^	CL11^a^	ZD808^a^	MPH (100%)	BPH (100%)
Spikelet differentiation stage	ear length (mm)	8.25 ± 1.07	6.4 ± 0.36	15.58 ± 0.69	112.7**	88.85**
	ear diameter (mm)	2.02 ± 0.19	1.42 ± 0.06	2.86 ± 0.32	66.28**	41.58**
Floret differentiation stage	ear length (mm)	14.23 ± 0.55	10.3 ± 1.15	20.67 ± 1.53	68.53**	45.26**
	ear diameter (mm)	2.4 ± 0.05	1.87 ± 0.03	3.4 ± 0.17	59.25**	41.67**
Mature ear	ear length (cm)	20.28 ± 1.00	15.05 ± 0.26	26.05 ± 0.58	47.49**	28.48**
	ear diameter (cm)	4.68 ± 0.20	4.08 ± 0.17	5.60 ± 0.34	28**	19.78**
	ear row number	15.50 ± 1.00	12.50 ± 1.00	16 ± 0.00	14.29**	3.23
	kernel number per row	32.25 ± 1.71	21 ± 1.15	50.75 ± 4.03	90.61**	57.36**
	grain yield (kg/mu)	301 ± 12.5	230 ± 4.5	553 ± 26.1	108**	84**

** indicate significant differences at *P* < 0.01

^a^ Values are means ± standard deviation. MPH, midparent heterosis; BPH, Best parent heterosis

In this study, using RNA sequencing technology, we presented a global gene expression profile of immature ears of maize hybrid ZD808 and its parental lines during the spikelet and floret differentiation stages. We investigated the gene expression divergence, allele-specific expression patterns and the *cis*- and *trans*-regulatory mechanisms underlying maize ear heterosis, and we compared the gene expression and regulation between the two developmental stages. Our research provides a comprehensive perspective on the transcriptomic changes and their correlations with heterosis during maize ear development.

## Results

### Characterizing the ear traits of ZD808 and its parental lines

**Fig. 1:**
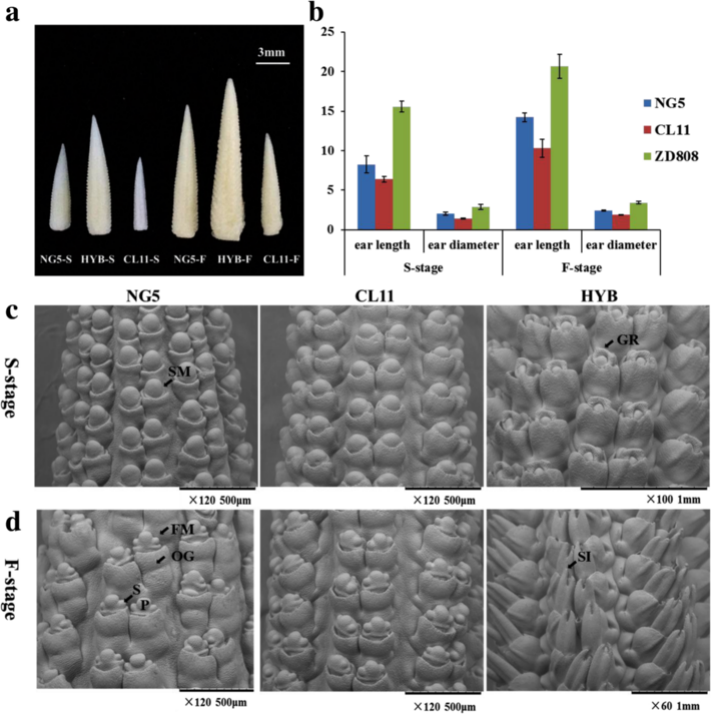
Characterization of the ear architectural traits of maize hybrid ZD808 (HYB) and its parental lines. **a** Phenotypic differences of the immature ears of HYB, CL11 and NG5 in the spikelet and floret differentiation stages. **b** Ear length and diameter of HYB, CL11 and NG5 in the spikelet and floret differentiation stages. Scanning electron microscope observation of immature ears of NG5, CL11 and HYB in spikelet (**c**) and floret differentiation stages (**d**). Abbreviations: S-stage, spikelet differentiation stage; F-stage, floret differentiation stage; SM, spikelet meristem; GR, Gynoecial ridge; FM, floret meristem; OG, outer glume; S, stamen primordia; P, pistil primordial; SI, silk

To dissect the relationship between global gene expression changes and heterosis during ear development, the immature ears of ZD808 (HYB) and its parent lines in spikelet and floret differentiation stages were collected for transcriptome analysis (Fig. 1a). In the spikelet differentiation stage (S-stage), the spikelet pair primordia (SPM) arise on the flanks of the inflorescence meristem (IM) and give rise to a pair of spikelet meristems (SM) (Fig. 1c). In the floret differentiation stage (F-stage), the SMs produce two floret meristems (FM) with obvious stamen and pistil primordia, (Fig. 1d). These two phases are crucial for ear development and heterosis formation. Further observation revealed that ear development in hybrids was more vigorous than in the parental lines, and the paternal line NG5 showed larger ear size than the maternal line CL11 (Fig. 1a and b). Significant mid-parent heterosis (MPH) and best-parent heterosis (BPH) (*p* < 0.01) were discovered for ear length and ear diameter in the S- and F-stages, and the MPH and BPH values were higher in the S-stage (Table 1). Furthermore, we also observed significant MPH and BPH (*p* < 0.01) for the ear length, ear diameter, kernel number per row and grain yield at the mature stage (Table 1). These results indicate that ZD808 displays a strong hybrid vigor than both parents and the degree of heterosis for ear traits was larger in the spikelet differentiation stage.

### Deep sequencing and mapping of maize inbred and hybrid transcriptomes

cDNA libraries of immature ears of ZD808 and its parental inbred lines CL11 and NG5 in the spikelet and floret differentiation stages were prepared and sequenced on an Illumina HiSeq 2G platform. To increase the statistical power, two biological replicates of each sample were sequenced. After reads with low sequencing quality were filtered out, between 43.9 and 63.5 million 100-bp paired-end reads were obtained for each of the replicates and genotypes. Among these, about 80.7% (516 million) of the total paired reads were aligned to the B73 reference genome (ZmB73_RefGen_v3) and 77.9% were mapped to unique positions. As expected, more than 97% of reads mapped to protein-coding genes, and the others were distributed among introns (0.4–1.3%) and intergenic regions (1.2–1.7%) (Additional file 1). Uniquely mapped reads were used to estimate transcript levels. Expression values were expressed in units of RPKM (reads per kilobase per million reads mapped). Two biological replicates were highly correlated, with an average Pearson’s correlation coefficient of 0.99 (Additional file 2).

For all analyses performed in the study, only the protein-coding genes were included, which are a subset of 39,469 gene models obtained after excluding transposons, pseudogenes, contaminants, and other low-confidence annotations. A transcript is considered to be positively expressed only if its RPKM ≥ 1. Based on this criterion, 21,258 genes were transcribed in at least one sample, which accounted for 53.9% of maize protein-coding genes (Additional file 3). On average, 2760 and 4314 genes (13.3 and 20.8% of expressed genes) exhibited high (RPKM ≥ 50) and medium (20 ≤ RPKM < 50) expression levels, respectively, and low expression genes accounted for 66.0% of the expressed genes (RPKM ≤ 20). More genes were expressed in hybrids compared with CL11 and NG5 in both developmental stages (Additional file 3).

### Expression divergence between hybrid and inbred parents

To fully elucidate the gene expression divergence and its effect on heterosis, we performed pairwise comparisons between the hybrid and the parents. Differentially expressed genes (DEGs) were identified if the RPKM value of a gene was greater than or equal to 1 in at least one of the genotypes and if the adjusted *p*-value for FDR was less than 0.05. Using this significance threshold, in the spikelet differentiation stage, we identified 12,550 genes differentially expressed between the parental lines CL11 and NG5, which accounted for 54.48% of analyzed genes (23,038) (Table 2). The high expression divergence confirmed the large genetic distance between the parental lines. The number of upregulated gene numbers is similar to the number of downregulated genes (27.26% up vs. 27.22% down), which implies equal contributions of CL11 and NG5 to gene expression divergence (Table 2). Advanced comparisons between hybrids and parental lines revealed fewer DEGs than the comparison of the two parental lines. Between HYB and CL11, 8290 (35.98%) DEGs were identified, including 4310 (18.71%) upregulated and 3980 (17.28%) downregulated. Between HYB and NG5, only 4309 (18.70%) DEGs were discovered, including 10.18% upregulated and 8.52% downregulated (Table 2). These results show that the expression profile of the hybrid is more similar to the paternal line NG5 and more divergent from CL11. In addition, more genes were actively expressed in the hybrid. In the floret differentiation stage, similar differential expression statuses were also discovered; however, the DEGs from each comparison were decreased. There were 10,120 (43.93%) differences between CL11 and NG5, 7315 (31.75%) between HYB and CL11, and 2696 (11.70%) between HYB and NG5 (Table 2). Gene differential expression underlies the phenotype variation; decreasing DEGs in the F-stage appropriately explained the lower MPH and BPH values of relative ear traits in the F-stage compared to the S-stage (Table 1).

**Table 2:** Differentially expressed genes between hybrid and the parents in the spikelet and floret differentiation stages

Comparison Group	UP		Down		Total	
	Gene number	Percentage	Gene number	Percentage	Gene number	Percentage
CL11-S vs NG5-S	6280	27.26%	6270	27.22%	12550	54.48%
HYB-S vs CL11-S	4310	18.71%	3980	17.28%	8290	35.98%
HYB-S vs NG5- S	2346	10.18%	1963	8.52%	4309	18.70%
CL11-F vs NG5-F	4985	21.64%	5135	22.29%	10120	43.93%
HYB-F vs CL11-F	4048	17.57%	3267	14.18%	7315	31.75%
HYB-F vs NG5-F	1475	6.40%	1221	5.30%	2696	11.70%
CLl1-S vs CL11-F	608	2.64%	366	1.59%	974	4.23%
NG5-S vs NG5-F	152	0.66%	288	1.25%	440	1.91%
HYB-S vs HYB-F	133	0.58%	352	1.53%	485	2.11%

Total analyzed genes: 23038; Deseq *P* value with padj < 0.05; S denotes the spikelet differentiation stage; F denotes the floret differentiation stage

Further comparison of the two development stages revealed 974, 485, and 440 DEGs in CL11-S vs. CL11-F, HYB-S vs. HYB-F, NG5-S vs. NG5-F, respectively (Table 2). Venn diagram showed that 249 genes were differentially expressed between two development stages in at least two genotypes (Additional file 2: Figure S2A). Hierarchical clustering and gene ontology (GO) enrichment revealed that these genes exhibited similar expression pattern among genotypes and mainly enriched ([FDR] < 0.01, Yekutieli FDR dependency) in ‘biological regulation’ and ‘developmental process’ (Additional file 2: Figure S2B and S3). Under these two categories, 52 (26.1%) and 28 (14.1%) DEGs, respectively, were identified to be involved in regulation of ear development, including some well-known genes (Additional file 4). *RAMOSA1* (*RA1*), *RAMOSA2* (*RA2*), *RAMOSA3* (*RA3*) and *BRANCHED SILKLESS*1 (*BD1*) control the determinacy and identity of the spikelet-pair meristem in maize, and they were upregulated in the spikelet differentiation stage in at least two genotypes [39–42]. *BARREN INFLORESCENCE2* (*BIF2*) together with *TEOSINTE BRANCHED 1* (*TB1*) affecting the initiation and maintenance of axillary meristems were also found upregulated in S-stage [43, 44]. Furthermore, *ZEA FLORICAULA*/*LEAFY*1 (*ZFL1*), *ZFL2 and DELAYED FLOWERING1 (DLF1)* genes which are required for floral transition also exhibited increased transcript levels in the S-stage [45, 46]. While in the floret differentiation stage (F-stage), MADS box (ZMM6, ZMM7, ZMM17, ZMM18 and ZMM29), GATA and C2C2-YABBY transcription factors which are crucial for floral meristem determinacy and organ development were significantly upregulated [47–49]. EREBP-transcription factors, Auxin efflux carrier component, Aux/IAA and Auxin response factor proteins which play key roles in gibberellin and auxin response during floral meristems initiation also increased expression abundance in F-stage (Additional file 4). These results confirm the validity of our chosen development stages.

**Fig. 2:**
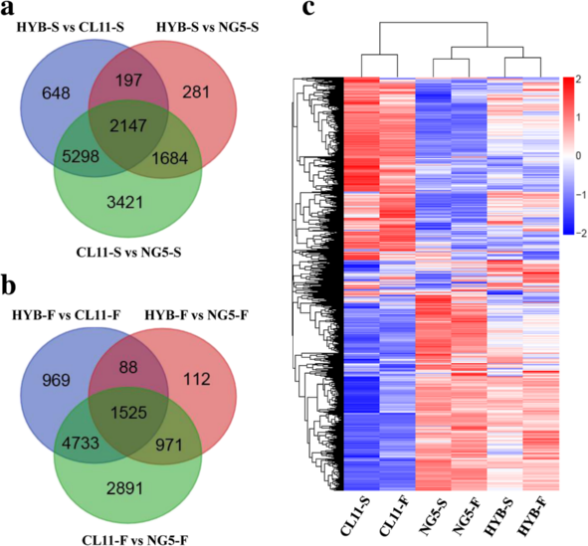
Venn diagram comparison and hierarchical cluster analysis of differentially expressed genes among genotypes. Venn diagram comparison of differential expressed genes between the hybrid and its parents in the spikelet (**a**) and floret differentiation stages (**b**) of maize immature ear. **c** Hierarchical cluster analysis of differentially expressed genes among genotypes. The color key represents log_10_(RPKM + 1). Red indicates high relative expression and blue indicates low relative expression. S denotes the spikelet differentiation stage, F denotes the floret differentiation stage

Using a Venn diagram to compare DEGs between hybrid and its parents reveals that 59.3% (5298 and 2147) and 61.8% (4733 and 1525) DEGs between CL11 and NG5, respectively, are differentially expressed between HYB and CL11 in the spikelet and floret differentiation stages, whereas only 30.5% (1684 and 2147) and 9.6% (971 and 1525) are differentially expressed between HYB and NG5. A total of 24.7% (2147 of 12,550) and 15.1% (1525 of 10,120) of DEGs were shared by the three comparisons (Fig. 2a and b). The results show that DEGs are common existence and differ among genotypes and developmental stages. Hierarchical clustering of DEGs showed that different developmental stages of the same genotype tend to cluster together, and the gene expression patterns in the hybrid were more similar to those in the paternal line NG5 in both developmental stages (Fig. 2c). This result corresponds to the more robust ear phenotype of NG5 observed at corresponding stages (Fig. 1a and b).

### Differential gene expression patterns and heterosis

To gain overall insight into differential gene expression patterns between the F1 hybrid and its parental lines, the DEGs from two developmental stages were further classified into additive and non-additive patterns based on the pairwise comparisons between expression levels of the hybrid and the mid-parent expression value (MPV). We identified 10,093 (73.8%) and 9014 (79.9%) genes expressed additively in the spikelet and floret differentiation stages, respectively, whereas only 3577 (26.2%) and 2272 (20.1%) displayed a non-additive expression pattern (Table 3). The prevalence of additively expressed genes implied complementary effects on gene expression in hybrid. According to the pairwise comparison results among CL11, NG5 and HYB, the non-additively expressed genes can be further divided into (I) over-dominance, (II) high-parent dominance, (III) low-parent dominance, (IV) under-dominance and (V) conserved expression classes. The detailed gene proportions of each class of two development stages are shown in Table 3. In the spikelet differentiation stage, the parent-dominant expression classes II and III accounted for the majority (2938, 82.14%) of non-additively expressed genes; 1981 genes (55.38%) exhibited NG5-dominant expression, and 957 genes (26.75%) exhibited CL11-dominant expression. Only 143 (4.0%) and 119 (3.3%) genes showed over-dominance (class I) and under-dominance (class IV), respectively (Table 3 and Additional file 5). In the floret differentiation stage, 1970 genes exhibited parent-dominant expression (II and III, 86.71% of 2272 non-additive expressed genes), with 1602 (70.51%) having NG5-dominant expression and 368 (16.20%) showing CL11-dominant expression. A total of 109 and 27 genes displayed over-dominance and under-dominance expression patterns, respectively (Table 3 and Additional file 6). These results revealed that the majority of DEGs displayed an additive expression pattern, suggesting that a complementary effect have a fundamental role in the early formation of maize ear heterosis. Among non-additively expressed genes, a significant number of genes showed an NG5-dominant expression pattern, implying that the NG5 allele may greatly affect the gene expression levels in hybrid and also contribute to hybrid vigor.

**Table 3:** Classification of additive and non-additive expression patterns in hybrid

Expression classes		S-Stage	F-Stage
Additive^a^ (MPV = F_1_)		10093 (73.8%)	9014 (79.9%)
Non-additive^a^(MPV ≠ F_1_)		3577 (26.2%)	2272 (20.1%)
I	Over-dominance ^c^	143	109
II High-parent dominance	CL11-dominance ^b^	492	227
	NG5-dominance ^b^	995	865
III Low-parent dominance	CL11-dominance ^b^	465	141
	NG5-dominance ^b^	986	737
IV	Under-dominance ^d^	119	27
V	Conserved^e^	377	166
Total		13670	11286

^a^based on fisher exact test between midparent value (MPV) and hybrid (qvalue < 0.05)

^b^based on fisher exact test (qvalue < 0.05); hybrid must be significantly different than midparent value and not significantly different from either high or low parent

^c^above high parent; based on fisher exact test between high parent and hybrid (qvalue < 0.05)

^d^below low parent; based on fisher exact test between low parent and hybrid (qvalue < 0.05)

^e^based on fisher exact test (qvalue < 0.05); hybrid value must be significantly different than midparent value and within the parental range

**Fig. 3:**
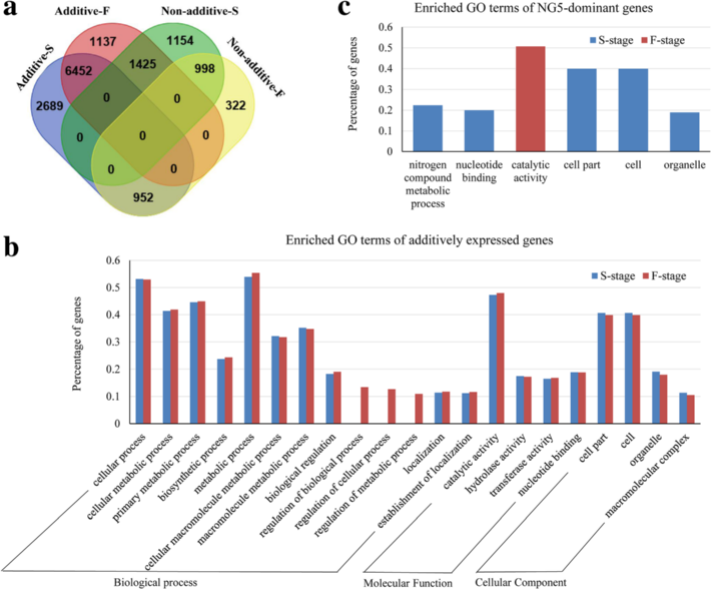
Comparison and functional enrichment of additive and non-additive genes in hybrids at different developmental stages. Comparison of additive and non-additive genes in hybrids at the spikelet and floret differentiation stages by venn diagram (**a**). Gene Ontology enrichment of additive genes (**b**) and NG5-dominant genes (**c**) in hybrids at the spikelet and floret differentiation stages

Gene expression profiles fluctuated with developmental stage, and comparisons of gene expression patterns between the two development stages revealed that 6452 (63.9%) additive genes from the spikelet differentiation stage maintained their additive expression status in the floret differentiation stage, whereas 952 genes changed to exhibit a non-additive expression pattern. In contrast, 998 (27.9%) of non-additive genes maintained the expression status in the floret differentiation stage, and 1425 (39.8%) genes changed to exhibit an additive expression pattern (Fig. 3a), which indicates that non-additively expressed genes were more affected by the development stage.

### Gene Ontology (GO) analysis of additive and non-additive genes

To ascertain the molecular and biological functions of genes with additive and non-additive expression patterns and to determine their biological roles in ear heterosis, we conducted GO enrichment analysis using single enrichments analysis from AgriGO website. In total, 6003 of 10,093 and 5350 of 9014 additively expressed genes in the spikelet and floret stages, respectively, were found to be enriched ([FDR] < 0.01, Yekutieli FDR dependency) in GO terms from biological process (BP), molecular functions (MF) and cellular component (CC). In the BP category, the most overrepresented subcategories were ‘metabolic process’ (54.0 and 55.4%), ‘cellular process’ (53.2 and 53.0%) and ‘biological regulation’ (18.3 and 19.1%) in both developmental stages. Further examination of specific subcategories in ‘metabolic process’ and ‘cellular process’ revealed that ‘primary metabolic process’ (44.6 and 44.9%), ‘cellular metabolic process’ (41.4 and 41.9%), ‘macromolecule metabolic process’ (35.2 and 34.7%) and ‘biosynthetic process’ (23.7 and 24.3%) were enriched in both sets of transcripts from the two stages. In the MF category, ‘catalytic activity’ (47.3 and 48.0%) and ‘nucleotide binding’ (18.9 and 18.9%) were prominently represented, while ‘cell’ (40.6 and 39.9%) and ‘organelle’ (19.2 and 18.0%) dominated the cellular component category (Fig. 3b and Additional file 7). These results revealed that genes from the additive expression class participated in extensive biosynthetic and metabolic activities, which are required for maize ear development and early heterosis formation in both development stages. Some classical genes such as *ZAG3* (GRMZM2G160565), *SID1* (GRMZM2G176175), *ZMM8* (GRMZM2G102161), and *ZMM14* (GRMZM2G099522), which affect the fundamental formation of maize ear architecture, were also expressed additively in both stages (Additional files 5 and 6).

In contrast, non-additively expressed genes were enriched in different GO functional categories in the spikelet and floret differentiation stages. In the spikelet differentiation stage, the CL11-dominant genes were overrepresented in BP terms such as ‘metabolic process’ (65.6%) and ‘cellular process’ (60.0%). The NG5-dominant genes were significantly enriched in the BP term ‘nitrogen compound metabolic process’ (22.4%) and the MF term ‘nucleotide binding’ (20.0%) (Fig. 3c and Additional file 7). Genes from the over-dominance expression class were found to be enriched in the MF term ‘hydrolase activity’ (29.7%), and no GO term was significantly enriched in genes from the under-dominance expression class. In the floret differentiation stage, only the NG5-dominant genes were significantly enriched in the MF term ‘catalytic activity’ (50.7%) (Fig. 3c and Additional file 7). More functional GO terms were enriched in the first stage, suggesting that non-additive genes played an important role during the period when spikelet pair meristems (SPMs) give rise to spikelet meristems (SMs).

We further inspected the NG5-dominant genes due to their high representation in the non-additive expression class. In the spikelet differentiation stage, many transcription factors, including MADS-box (e.g., *ZGA5*, *SI1*), bHLH DNA-binding superfamily protein, Auxin response factor and Ethylene-responsive transcription factor were found in the “cellular process” GO term, which may trigger cell identity, regulate hormone signaling and promote the transition from SPMs to SMs. In the “nitrogen compound metabolic process” term, 35 genes were discovered that participate in glutamine synthesis (e.g., *GLN4*), glutamine metabolism and aspartate metabolism, which are essential for nitride assimilation and affect maize ear development and spikelet formation (Additional file 8). In the floret differentiation stage, 36 genes participated in oxidation-reduction reactions (e.g., *GA20OX1*, *APX2, CAT1,* and *CAT3*) and were found in the “catalytic activity” term, suggesting a role in stress responses and signal transduction in the floret differentiation stage (Additional file 8).

### Global allele-specific expression (ASE) analysis

**Fig. 4:**
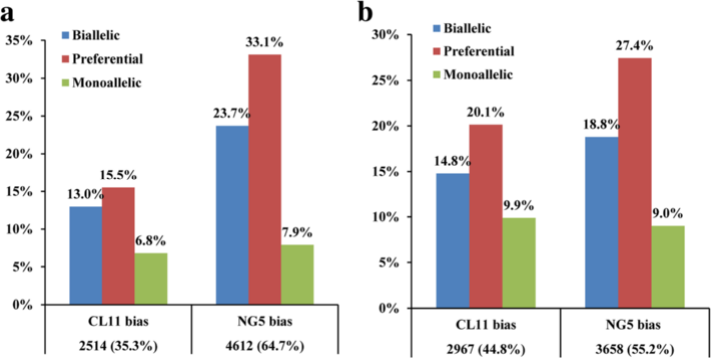
The allele-specific expression analysis in hybrid in the spikelet and floret differentiation stages. The proportions of genes with monoallelic expression, preferential allelic expression and biallelic expression profiles in hybrid in the spikelet (**a**) and floret differentiation stage (**b**) of maize ear

The transcriptional activities of different alleles in a hybrid can differ considerably, and this is an important source of the variation in gene expression. To infer hybrid ASE levels, parent-specific SNPs were detected in each parent and used to discern alleles in the hybrid. After applying quality control criteria, we found that 44,675 and 38,957 of SNPs located in gene bodies had a minimum read coverage of 10 in the hybrid at the spikelet and floret differentiation stages, respectively. A total of 12,637 and 11,993 genes, which represented 32.0 and 30.4% of protein coding genes, respectively, were marked by the filtered SNPs (Table 4). Normalized mapped read-depth coverage at SNP sites in the hybrid and parental alignments was used to quantify the expression of alleles. Allelic bias in the hybrid was identified for each SNP if the allelic ratio differed significantly from the expected allelic ratio of 1.0 (binomial exact test, adjust *p*-value < 0.05). For convenience, we used CL11_HYB_ and NG5_HYB_ to represent the expression levels of the corresponding allele in the hybrid. In the spikelet differentiation stage of maize ear, 7126 genes (56.4% of 12,637 analyzed genes) were identified as having significant allelic bias. Of these, 2514 (35.3%) genes displayed CL11_HYB_ bias and 4612 (64.7%) genes displayed NG5_HYB_ bias (Fig. 4 and Additional file 9). In the floret differentiation stage, we identified 6625 (52.4%) ASE genes; 2967 (44.8%) genes exhibited CL11_HYB_ bias and 3658 (55.2%) genes exhibited NG5_HYB_ bias (Fig. 4 and Additional file 10). These results indicated a strong expression bias toward NG5_HYB_ in both developmental stages, suggesting that the NG5 genome contributes greatly to gene expression in the hybrid.

**Table 4:** SNPs for assessing allele-specific gene expression in hybrid in the spikelet and floret differentiation stages

	SNPs for ASE analysis	Reads covered SNPs	Reads/SNP	Gene numbers	SNPs/Gene	% of protein coding gene
S-Stage	44675	3113870	69.7	12637	3.5	32.0
F-Stage	38957	2647720	68.0	11993	3.2	30.4

According to the extent of bias, the ASE genes can be classified into three different classes: monoallelic expression (only one allele is expressed in the hybrid), preferential expression (expression level differs by more than two-fold between CL1_HYB_ and NG5 _HYB_) and biallelic expression (expression level varies by less than two-fold between CL11_HYB_ and NG5_HYB_). Using these criteria, we found that 36.7 and 33.6% of ASE genes had biallelic expression, 48.6 and 47.5% showed preferential allelic expression, and 14.7 and 18.9% had monoallelic expression in the spikelet and floret development stages, respectively, which further confirmed the substantial expression preferences of parental alleles in the hybrid (Fig. 4 and Additional files 9 and 10).

Comparison of ASE in the two developmental stages revealed that among the 10,388 genes analyzed in both stages, 7261 (69.9%) genes exhibited conserved allele bias patterns in the two stages, while 495 CL11_HYB_ biased genes and 1244 NG5_HYB_ biased genes changed their expression bias. There were also 1388 genes with non-biased expression that were converted into biased allelic expression patterns (Additional file 11). These results indicate that ASE varies between developmental stages, and suggest the presence of *cis*-regulatory elements interacting with development cues.

### *Cis* and *trans* effects on gene expression divergence

**Fig. 5:**
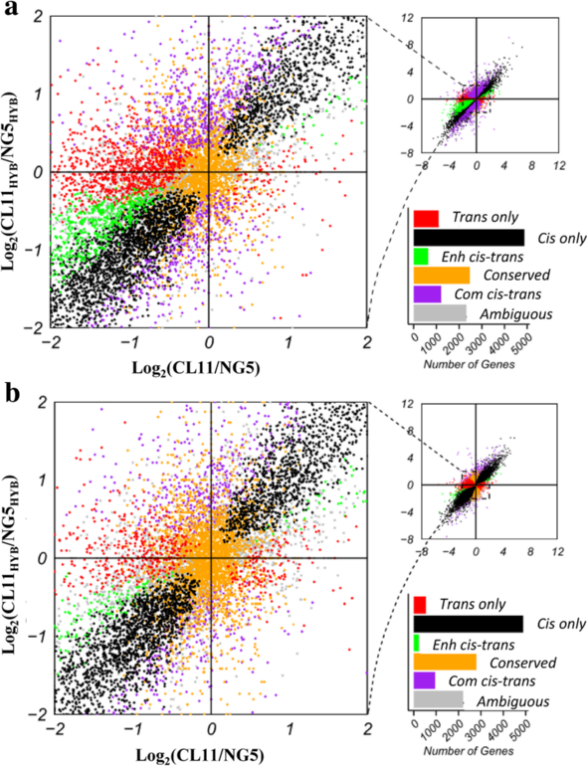
The Plot summarizes the relative allele-specific expression levels in parental and F1 hybrids. Each point represents a single gene and is color-coded according to the mechanism of regulatory evolution inferred from statistical tests. The bar graph depicts the number of genes in each category in the spikelet (**a**) and floret differentiation stage (**b**) of maize ear

In the hybrid, gene expression divergence between parental alleles can result from changes in *cis*- and/or *trans*-regulation [30, 50]. Therefore, we compared ASE in the parental lines and the hybrid in the spikelet and floret differentiation stages to identify *cis*-and *trans*-regulation divergence. The expression difference between the two parental inbred lines reflected both *cis* and *trans* effects. In the hybrid, because both alleles were under the same genetic background and shared a common set of *trans*-regulatory factors, the allelic expression divergence in the hybrid was considered to represent *cis* effects. *Trans* effects could be detected by subtracting *cis* effects from the overall set of *cis* and *trans* effects. According to this classification criterion, in the spikelet differentiation stage, we identified 4869 (38.6%) and 1107 (8.8%) genes that showed *cis* only and *trans* only effects, respectively, whereas 1868 (14.8%) genes were associated with both *cis* and *trans* effects (*cis-trans*). Over two thousand (2476, 19.6%) genes showed no significant evidence of either parental expression divergence or significant *cis*-or *trans*-regulation divergence and were classified as “conserved.” A total of 2317 (18.3%) genes had an “ambiguous” expression pattern with no clear biological interpretation (Fig. 5a and Additional file 12). The results showed that there were significantly more *cis*-regulatory effects than *trans*-regulatory effects. The 1868 genes with both *cis* and *trans* effects were further subdivided into two categories based on the acting direction of the *cis* and *trans* effects: “enhancing,” in which both the *cis*-and *trans*-regulation effect favored expression of the same allele, and “compensating,” in which both the *cis*- and *trans*-regulation effect favored expression of the opposite allele. Among the 1868 genes, 652 genes (34.9%) were subjected to enhancing *cis* and *trans* interactions and 1216 genes (65.1%) were related to compensating *cis* and *trans* interactions (Fig. 5a and Additional files 12 and 13). In the floret differentiation stage, among the 11,993 analyzed genes, 4888 (40.8%), 549 (4.6%), 235 (19.7%) and 960 (80.3%) genes fell into the *cis* only, *trans o*nly, “enhancing” and “compensating” *cis*-*trans* interactions, respectively, and 3156 genes were classified as “conserved.” The remaining 2205 genes showed an “ambiguous” expression pattern and were excluded from further analysis (Fig. 5b and Additional files 12 and 14). Detailed analysis showed that a large number of *cis* and *trans* genes exhibited negative log_2_ ratios of allelic expression divergence in both development stages, which implies that both *cis*- and *trans*-regulatory divergence tend to cause a higher expression of the NG5 allele (Additional file 12). This finding is consistent with the high level of NG5_HYB_ allelic bias in the hybrid.

Comparisons between the two developmental stages revealed that, among the 10,388 genes analyzed in both developmental stages, 65.5% of the *cis* only genes from the spikelet stage maintained a consistent regulation pattern in the floret stage, but merely 17.8% of the *trans* only genes and 26.6% of the *cis-trans* interaction genes maintained their regulation patterns (Additional file 15).

### Relationship between *cis-* and *trans-*regulatory divergence and gene expression patterns

**Fig. 6:**
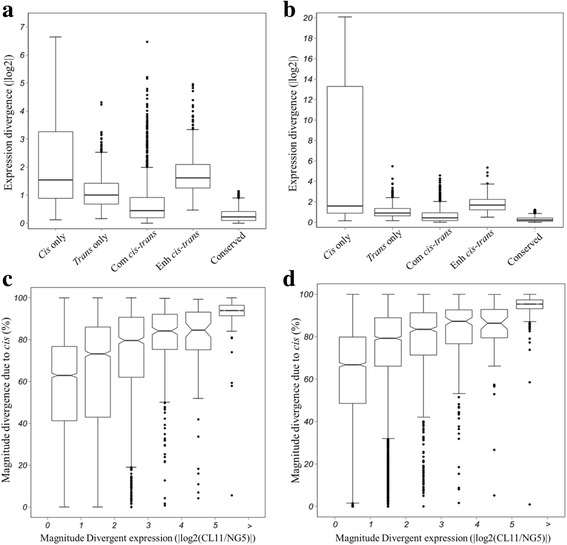
The relative contributions of *cis*- and *trans*-regulation to variation in gene expression. Absolute magnitude (fold-change) of parental divergence resulting from *cis* only, *trans* only, compensating (Com) and enhancing (En) *cis* and *trans* interaction in the spikelet differentiation stage (**a**) and floret differentiation stage (**b**) of maize ear. Box-and-whisker plots showing the percent of *cis*-effects for genes binned based on the magnitude of expression divergence between parents in the spikelet differentiation stage (**c**) and floret differentiation stage (**d**) of maize ear

Finally, we looked for correlations between gene expression patterns and the mechanism of regulatory divergence. The absolute magnitude of parental divergence resulting from different regulation categories illustrated that a vast majority of gene expression differences between parents were regulated by *cis* effects (Fig. 6a and b), and the proportion of total expression divergence regulated by *cis* effects increased with the magnitude of divergence (Fig. 6c and d).

**Fig. 7:**
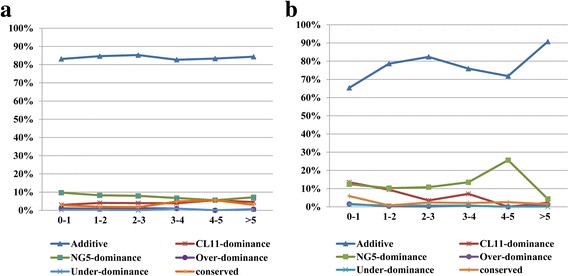
The percentage of *cis*-effects for genes showing additive and non-additive inheritance. Distributions of percent *cis* for genes showing additive, CL11-dominance, NG5-dominance, over-/under-dominance and conserved inheritance in the spikelet differentiation stage (**a**) and floret differentiation stages (**b**) of maize ear. Total divergence (log_2_ of parent expression ratio) was binned from 0–1, 1–2, 2–3, 3–4, 4–5 and >5

Genes subject to *cis*-regulatory variation are expected to have additive effects on gene expression in the hybrids [31]. We compared the proportions of genes showing additive and non-additive gene action in the *cis* only list. More than 65% of *cis* genes exhibited additive gene action in the spikelet and floret stages, and 84 to 91% of *cis* genes contributed to an additive expression pattern when the log_2_ ratio of parent expression divergence was greater than 5. Only a few *cis* genes displayed a dominant and over/under-dominant expression pattern (Fig. 7a and b).

### SNP confirmation and qRT-PCR validation of differentially expressed genes from RNA-seq

**Fig. 8:**
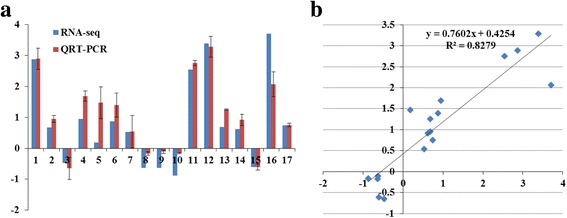
Correlation between qRTPCR and RNA-seq with selected differentially expressed genes. **a** qRT-PCR validation of differentially expressed genes in the spikelet and floret differentiation stages from RNA-seq. **b** Correlation analysis in log2 fold change measurement between RNA-seq and qRT-PCR.

To confirm the accuracy and reproducibility of the RNA-seq results, 17 differentially expressed genes were randomly selected for real-time PCR (qRT-PCR), and the correlation between RNA-seq and qRT-PCR was evaluated using log_2_-fold change measurements. The qRT-PCR results showed that the expression trends of these genes were significantly similar (r^2^ = 0.83) to those of the RNA-seq data (Fig. 8 and Additional file 16). To verify the accuracy of SNPs used in ASE analysis, 115 SNPs from 35 genes were amplified from each genotype using gDNA and cDNA as PCR templates, and the PCR products were sequenced with the Sanger method. All the SNPs were confirmed correctly (Additional file 17).

## Discussion

### Prevalent differential gene expression among ZD808 and its parental lines

Maize ear inflorescence development is a complex and dynamic biological process involving different regulatory networks and a large number of genes. Our RNA sequencing results revealed that 21,258 genes (53.9% of maize protein coding genes) were transcribed in at least one genotype (Additional file 3). This finding indicates the active gene regulation underlying immature ear development. More genes were expressed in the hybrid compared to CL11 and NG5 in both developmental stages, suggesting that complementation contributes to transcriptome complexity in hybrids and helps explain hybrid phenotypic advantages.

Previous studies have suggested a correlation between heterosis and gene expression variation. Substantial numbers of differentially expressed genes were discovered among the hybrids and their parents in many species [15, 17, 31, 51]. Using microarray technology, 4–18% of expressed genes were identified as being significantly differentially expressed in different genetic backgrounds of maize immature ear, seedling and embryo tissues [15, 31]. In rice, 10.6% of the total gene set is differentially expressed in the super hybrid LYP9 and its parental cultivars 93–11 and PA64s [51]. RNA-seq technology revealed higher proportions of DEGs among the cultivars Nipponbare, 93–11 and their reciprocal F1 hybrids [17]. A recent report revealed that nearly 70% of maize expressed genes were differentially expressed between B73 and Mo17, and 42–55% were differentially expressed between hybrids and their parents [20]. In our study, 54.5 and 43.9% DEGs were identified between CL11 and NG5 in the spikelet and floret differentiation stages of maize ear (Table 2). The relatively high percentages of DEGs between CL11 and NG5 demonstrated the large genetic distance between the two parental lines, which may be an important reason for the superior performance of ZD808. Analysis of gene expression differences between the hybrid and its parental lines revealed that more DEGs occurred between the hybrid and CL11 (36.0 and 31.8%) compared with the DEGs between the hybrid and NG5 (18.7 and 11.7%) in both developmental stages (Table 2). These data suggest that gene expression in the hybrid is more similar to the paternal line NG5. This result is further confirmed by hierarchical clustering analysis (Fig. 2c). Observation of the ear phenotypes of CL11 and NG5 showed that significant MPH and BPH were observed in both development stages, and the paternal line NG5 was more vigorous than CL11 (Fig. 1a and b and Table 1). Thus, we can further deduce that NG5 may play an important role in the ear heterosis of ZD808.

In the spikelet differentiation stage, the MPH and BPH values of the ear length and ear diameter were comparatively higher compared to those in the floret differentiation stage (Table 1). Comparisons between the two developmental stages revealed more DEGs between ZD808 and its two parents in the spikelet differentiation stage, which implies that the DEGs may be positively correlated with the degree of heterosis.

### Additive gene expression patterns play fundamental roles in maize ear heterosis

The debate regarding the relationship between heterosis and additive and non-additive gene expression has been discussed in previous studies. Using immature ear tissues of a series of 16 maize hybrids as materials, Guo et al. revealed that the proportion of additively expressed genes is positively associated with hybrid yield and heterosis, whereas non-additively expressed genes are negatively correlated or not correlated with either yield or heterosis [14]. Swanson-Wagner et al. reported that only ~25% of differentially expressed maize genes exhibited non-additive expression profiles; the vast majority of them were expressed within the range of the two parents [31]. Similar findings were also reported by Stupar et al., who found no obvious correlation between non-additive expression and different heterosis levels [16]. Using RNA-seq, Paschold et al. discovered that only 10% of analyzed genes were non-additively expressed [20]. However, in other studies, genes with dominant or transgressive expression were more prevalent and considered to be important in conferring novel or superior hybrid performance. Auger et al. revealed that a substantial number of genes are not expressed at the mid-parent level in maize hybrids [24]. In maize internodes, over 50% of expressed genes showed an over-dominant gene expression pattern, and only 10.2% showed additive gene action [21]. A recent study of nascent allohexaploid wheat revealed that a high proportion of protein-coding genes exhibit parental expression level dominance and contribute to growth vigor [19]. In certain other species, transgressive expression seems to be especially common [32, 52, 53].

Consistent with previous studies, our results support the notion that multiple gene expression patterns exist between ZD808 and its parental lines. Among all the gene expression models, 73.8 and 79.8% of genes were additively expressed in the hybrid (Table 3), which suggests that the complementary interaction of two parental alleles occurred in the hybrid for most genes. This complementary effect might neutralize the effect of deleterious alleles and adjust the gene expression level into an optimal status. Springer et al. posited a similar hypothesis that the mid-parent expression pattern may increase the fitness in hybrid and play a foundation role in heterosis [25]. In addition, 26.2–20.1% of genes displayed non-additivity expression pattern. Of these, more than 80% were expressed at parental-like levels, and 55.4–70.5% were NG5-dominant genes (Table 3). These results suggest that the NG5 may provide many advantageous alleles that confer specific functions in maize ear development and lead to heterosis.

GO enrichment analysis revealed that additively expressed genes were mainly overrepresented in basic biological process and molecular functions in both development stages. In the biological process category, ‘metabolic process’ (54.0 and 55.4%), ‘cellular process’ (53.2 and 53.0%) and ‘biological regulation’ (18.3 and 19.1%) were the most highly represented GO categories. Under the ‘metabolic process’ and ‘cellular process’ GO terms, ‘primary metabolic process’ (including ‘carbohydrate metabolic process’ and ‘protein metabolic process’), ‘cellular metabolic process’ (including ‘cellular macromolecule metabolic process’ and ‘cellular biosynthetic process’), ‘macromolecule metabolic process’ (including “protein metabolic process’, ‘gene expression’ and ‘macromolecule biosynthetic process’) and ‘biosynthetic process’ (including ‘cellular biosynthetic process’ and ‘cell macromolecule biosynthetic process’) were found enriched in both stages (Fig. 3b Additional file 7). These results indicated that additively expressed genes were functioned in carbohydrate, protein and cellular macromolecule biosynthesis and metabolism in both stages, which are essential processes that produce both structural components and energy sources for maize ear development and heterosis formation.

While the non-additive expressed genes differently enriched between spikelet and floret differentiation stages. Among the NG5-dominant genes, the BP terms ‘nitrogen compound metabolic process’ (22.4%) and the MF terms ‘nucleotide binding’ (20.0%) were significantly enriched in the spikelet differentiation stage, but only “catalytic activity” (50.7%) was enriched in the floret differentiation stage (Fig. 3c and Additional file 7). Further inspection of the NG5-dominant genes revealed MADS-box, bHLH DNA-binding superfamily protein, Auxin response factors and Ethylene-responsive transcription factors in the ‘cellular process’ category (Additional file 8), these genes play important roles in coordinating the growth and differentiation of cells into new organs and regulating auxin or ethylene signaling to promote the transition from SPMs to SMs. Nitrogen (N) is crucially required for maize ear development, and N deficiency may reduce the kernel number and decrease the grain yield [54]. Many genes were discovered involving in glutamine synthesis, glutamine metabolism and aspartate metabolism in the ‘nitrogen compound metabolic process’ term. These genes may be essentially important for nitrogen assimilation and affect maize ear development. In the floret differentiation stage, genes involved in oxidation-reduction reactions were found in the ‘catalytic activity’ term; these genes may mainly participate in stress responses and signal transduction (Additional file 8). These results indicated that additive expressed genes were fundamentally required for ear heterosis formation in both development stages, while NG5-dominant genes may contribute to the stage-specific vigor phenotype.

### Allele-specific expression contributes to differential gene expression

Allelic variation is widespread in the maize genome, and combinations of parental allelic variants in hybrids may result in novel patterns of gene expression and contribute to superior phenotypes. Allelic expression bias was consistently observed for 50 and 60% of genes assayed in maize hybrid seedlings and meristems [27, 55]. In the rice hybrid Xieyou9308, 17% of transcripts showed significant allelic bias at the tillering and heading stages [56]. A similar study of a reciprocal F1 hybrid between rice Nipponbare and 93–11 revealed that 22.7% of genes exhibited significant allelic expression differences [57]. In *Arabidopsis*, about 40% of genes showed allelic expression differences in the hybrid [58]. In our study, the global ASE profile of the ear of ZD808 indicates that 56.4 and 52.4% of analyzed genes exhibited significant allelic expression bias in the spikelet and floret differentiation stages, respectively (Fig. 4a  and b). The higher rate of ASE in our results suggests that a large number of allelic variations may exist between CL11 and NG5.

Our results also show that the majority of ASE genes (64.7 and 55.2%) displayed NG5_HYB_ allelic expression bias in both developmental stages (Fig. 4a and b). The results suggest that the NG5 genome contributes greatly to the activity of the transcriptomes in the hybrid and explains the high level of NG5-dominant expression. A comparison of the ASE pattern between the two development stages revealed that 30.1% of ASE genes in the spikelet differentiation stages changed their allelic bias pattern, which indicates that the parental alleles in hybrids may exhibit *cis*-regulatory variation that result in differential responses to development cues (Additional file 11).

### *Cis-* and *trans-*regulatory differences underlying gene expression novelty

Quantitative changes in allele-specific expression may be the result of *cis*- and/or *trans*-regulatory variations [28, 59]. In our research, in the spikelet and floret developmental stages, respectively, 38.5 and 40.8% of analyzed genes were affected by *cis* only regulatory divergence, 8.8 and 4.6% of expressed genes were affected by *trans* only effects, and 14.8 and 10.0% of genes showed evidence of both (Fig. 5a and b and Additional file 12). The prevalence of *cis*-regulatory variation reflects the frequency of allelic variation in the maize genome and suggests the maintenance of inbred allelic expression levels in the hybrid. We also found that both *cis*- and *trans*-regulation tend to drive higher expression of the NG5 alleles, which explains the high level of NG5_HYB_ bias in the hybrid and the NG5-dominant expression pattern. The *cis* and *trans* effect genes were further classified into enhancing and compensating interactions. Previous studies have revealed that stabilizing selection is characterized by compensating *cis* and *trans* effects and diversifying selection corresponds to enhancing *cis* and *trans* effects [34, 60]. In our study, 60.7 and 80.3% of *cis*- and *trans*-regulation genes, respectively, exhibited compensating interaction in both development stages (Fig. 5a and b and Additional file 12), which suggests a prevalent role for stabilizing selection in maintaining gene expression levels.

Further analysis of the absolute magnitude of parental divergence resulting from different regulation categories illustrated that the vast majority of gene expression divergences between parents were regulated by *cis* effects (Fig. 6a and b), and the proportion of total expression divergence regulated by *cis* effects increased withthe magnitude of divergence (Fig. 6c and d). Taken together, these data indicate that *cis*-regulatory effects play a larger role than *trans* effects. Some studies have found that *cis-*regulatory variation is positively correlated with additive expression patterns in the F1 hybrid [30, 31]. Our study found that 65 to 91% of *cis*-regulated genes exhibit additive gene activity in the spikelet and floret differentiation stages (Fig. 7a and b). These data provide evidence that prevalent *cis*-regulatory variations contribute to allelic expression bias and result in an additive expression pattern in the maize hybrid ZD808.

Between the two stages, only 65.5% of *cis* only genes, 17.8% of *trans* only genes and 26.6% of *cis*-*trans* interaction genes maintained a consistent regulatory pattern (Additional file 15), which suggests that developmental stage-specific *cis*-/*trans*-regulation may explain the differential gene expression patterns between the two stages and lead to different manifestations of ear heterosis. Previous studies have also reported that *cis*-/*trans*-regulated variation shows differential responses to environmental [30] or developmental signals [61, 62].

## Conclusion

Using RNA sequencing technology, we systematically investigated the global transcriptomes of maize ear from the hybrid ZD808 and its parents in the spikelet and floret differentiation stages. Our results demonstrated that additive gene expression patterns were prevalent in the hybrid in both development stages, which suggested that the complementary interaction of two parental alleles occurred in the hybrid for most genes. This complementary effect may adjust the gene expression level into an optimal status and play a foundation role in maize ear heterosis. Among the non-additively expressed genes in the hybrid, the majority of which were expressed at NG5 dominant level, indicating that paternal line NG5 may provide beneficial alleles contributing to hybrid vigor. Analysis of allele-specific expression patterns in the hybrid suggested that variation in gene expression levels was largely attributable to *cis*-regulatory variation in maize. The *cis*-regulatory variations tend to preserve the allelic expression levels in hybrid and cause additive expression. Comparison between the two stages revealed that allele-specific expression and *cis*-/*trans*-regulatory variations responded differently to developmental cues, which may lead to different degree of heterosis during maize ear development.

Therefore, our work provides a comprehensive insight into transcriptional variation and its correlation with heterosis during maize ear development. The findings improve our understanding of the molecular basis of heterosis in maize and present novel opportunity to improve our maize varieties in the future.

## Methods

### Sample preparation and transcriptome sequencing

ZD808 (HYB) and its parental lines CL11 and NG5 were grown in experimental fields in the spring of 2013 under regular farming conditions in Nanchong, Sichuan. Ears were manually collected at the spikelet and floret differentiation stages according to the leaf age index combined with scanning electron microscopy. Five immature ears were pooled for the two biological replicates per genotype and were ground in liquid nitrogen.

Total RNA was extracted from each sample by using TRIzol reagent (Invitrogen, CA, USA). RNA degradation and contamination were monitored on 1% agarose gels. RNA quality was assessed using the Bioanalyzer 2100 system (Agilent Technologies, CA, USA) with a minimum RNA integrity number (RIN) of 7.0.

A 3-μg RNA sample was used as input material for library preparation. Sequencing libraries were generated using the NEBNext® Ultra™ RNA Library Prep Kit for Illumina® (NEB, USA), and index codes were assigned to each sample. Library quality was assessed on the Agilent Bioanalyzer 2100 system. The clustering of the index-coded samples was performed on a cBot Cluster Generation System using the TruSeq PE Cluster Kit v3-cBot-HS (Illumina). After cluster generation, the prepared libraries were sequenced on an Illumina HiSeq 2000 platform and 100 bp paired-end reads were generated.

### Sequenced read processing, alignment and gene expression level quantification

The raw reads were filtered before data analysis by removing reads consisting of adaptors, reads with more than 10% N, and low-quality sequences (more than 50% of the reads having a phred base sQ ≤ 5). The paired-end clean reads were aligned to the B73 reference genome (RefGen_v3) using the default parameters of TopHat v2.0.9. Reference genome and gene model annotation files were downloaded from genome website (http://ensembl.gramene.org/Zea_mays/Info/Index) directly.

HTSeq v0.5.4p3 was used to count the read numbers mapped to each gene. The RPKM of each gene was calculated based on the length of the gene, and read counts were mapped to the gene.

### Identification and classification of differential gene expression patterns

Differential expression analysis was performed using the R package DESeq. For all comparisons, the resulting *p* values were adjusted using Benjamini and Hochberg’s approach for controlling the false-discovery rate. Genes that exhibited an adjusted *p*-value < 0.05 (q-value) were determined to be significantly differentially expressed. To gain overall insight into gene expression inheritance patterns in the F1 hybrids and parental lines, the gene expression levels in the hybrids were compared to the mid-parent expression level. Genes with q-value < 0.05 were regarded as non-additive. Genes with q-value > 0.05 were regarded as additive. The non-additive genes were further classified into more specific categories. Genes with an F1 genotype mean that was not significantly different from one parent but was significantly higher (or lower) than the other parent were considered to exhibit high-parent (low-parent) dominance. Genes with an F1 genotype mean that was significantly higher (or lower) than both of the inbred line parents were said to exhibit over-dominance (under-dominance).

### SNP identification and allele-specific expression analysis

Picard-tools v1.96 and SAMtools v0.1.18 were used to sort and mark duplicated reads and reorder the bam alignment results of each sample. GATK2 software was used to perform SNP calling. Unreliable SNPs between CL11 and NG5 were filtered out according to the following criteria: 1) all reads uniquely match both CL11 and NG5 genomes, and the read quality value is no lower than 20; 2) all reads from one parent produce a consensus base at the SNP position but different from another parent; 3) the SNP is represented by at least 10 reads. Allelic bias in hybrids was identified by determining for each SNP whether there was significant deviation from the binomial distribution of parental alleles (i.e., the allele ratio in the hybrids deviated from 1.0).

### *Cis-* and *trans-*regulatory effects

To estimate the relative contributions of *cis-* and *trans-*regulatory factors, we performed statistical tests to compare the ratios of expression of the two parental alleles in the hybrid with the relative expression level of the consistent allele in the parental lines, as described in a previous study [32]. The overall gene expression divergence was quantified as log_2_ (CL11/NG5) (A) using the binomial exact test, with the *p*-value corrected by the FDR method. The significant *cis-*effects, referred to as allelic imbalance in the hybrid, were also determined with the binomial exact test. The extent of the *cis-*effects was quantified as log_2_ (CL11_HYB_/NG5_HYB_) (B). Fisher’s exact test followed by FDR analysis was used to divide *trans-*effects by subtracting *cis-*effects from the expression divergence between CL11 and NG5 (A-B). Based on the significance test, the genes could be categorized into five classes: 1) *Cis* Only: significant expression differences in A and B; no significant A-B; 2) *trans* Only: significant expression in A but not B; significant A-B; 3) *cis* and *trans* effects (*cis-trans*): Significant differential expression in A and B; significant A-B; 4) Conserved: No significant differential expression in A or B; no significant A-B; 5) Ambiguous: All other patterns of significance tests, with no clear biological interpretation.

The *cis-trans* category can be further divided into Compensating and Enhancing *cis* and *trans* interactions. The Enhancing *cis-trans* interaction was implied if the log_2_-transformed allele-specific ratios of these genes in the parental and hybrid data sets had the same direction. If they were in the opposite direction, the interaction was Compensating.

## Additional files

Additional file 1: Table S1. RNA-seq data statistics and genome mapping rates. (XLSX 16 kb)
Additional file 2: Figure S1. Pearson’s correlation coefficient between two biological replicates of each genotype calculated using log_10_(RPKM + 1). **Figure S2.** Analysis of genes differentially expressed between the spikelet and floret differentiation stages in three genotypes. **Figure S3.** Directed Acyclic Graph (DAG) visualization for Gene Ontology enrichment of 249 differentially expressed genes. (DOCX 1694 kb)
Additional file 3: Table S2. Distribution of gene expression levels in each genotype. (DOCX 14 kb)
Additional file 4: Table S3. DEGs involved in regulation of ear development from the 'developmental process' and 'biological regulation' terms. (XLSX 16 kb)
Additional file 5: Dataset S1. The differential gene expression patterns in the spikelet differentiation stage. (XLSX 3916 kb)
Additional file 6: Dataset S2. The differential gene expression patterns in the floret differentiation stage. (XLSX 3226 kb)
Additional file 7: Table S4. Cross-comparison of enriched GO terms of additive and non-additive expressed genes in the spikelet and floret differentiation stages. (XLSX 30 kb)
Additional file 8: Table S5. The selected NG5-dominant expressed genes that were assigned to different GO terms in the spikelet and floret differentiation stages. (DOCX 20 kb)
Additional file 9: Dataset S3. ASE patterns in hybrid in the spikelet differentiation stage. (XLSX 1350 kb)
Additional file 10: Dataset S4. ASE patterns in hybrid in the floret differentiation stage. (XLSX 1280 kb)
Additional file 11: Table S6. Comparison of the ASE patterns in hybrid between the two development stages. (DOCX 16 kb)
Additional file 12: Table S7. Gene numbers of different *cis*- and *trans*-regulatory categories. (DOCX 15 kb)
Additional file 13: Dataset S5.
*Cis*- and *trans*-regulatory variations in the spikelet differentiation stage. (XLSX 1351 kb)
Additional file 14: Dataset S6.
*Cis*- and *trans*-regulatory variations in the floret differentiation stage. (XLSX 1280 kb)
Additional file 15:Table S8. Comparison of the *cis*- and *trans-* regulatory variations between the spikelet and floret differentiation stages. (DOCX 14 kb)
Additional file 16: Table S9. qRT-PCR validation of differentially expressed genes in the spikelet and floret differentiation stages from RNA-seq. (DOCX 20 kb)
Additional file 17: Table S10. Verified SNPs locus in ASE analysis. (DOCX 37 kb)

## Abbreviations

ASE: Allele-specific expression
BPH: Best-parent heterosis
DEGs: Differentially expressed genes
FDR: False discovery rate
FM: Floret meristem
F-stage: Floret differentiation stage
GO: Gene ontology
IM: Inflorescence meristem
MPH: Mid-parent heterosis
RNA-Seq: RNA sequencing technology
RPKM: Reads per kilobase per million reads
SM: Spikelet meristem
SNP: Single-nucleotide polymorphism
SPM: Spikelet pair primordia
S-stage: Spikelet differentiation stage
