# Supplementary material for: Transcriptome profiling and comparison of maize ear heterosis during the spikelet and floret differentiation stages
Source: BMC Genomics. 2016 Nov 22;17:959. doi: 10.1186/s12864-016-3296-8 (PMC5120533; doi:10.1186/s12864-016-3296-8)
Supplement: Additional file 2: Figure S1. — Pearson’s correlation coefficient between two biological replicates of each genotype calculated using log10(RPKM + 1). Figure S2. Analysis of genes differentially expressed between the spikelet and floret differentiation stages in three genotypes. Figure S3. Directed Acyclic Graph (DAG) visualization for Gene Ontology enrichment of 249 differentially expressed genes. (DOCX 1694 kb) [file 12864_2016_3296_MOESM2_ESM.docx]

**
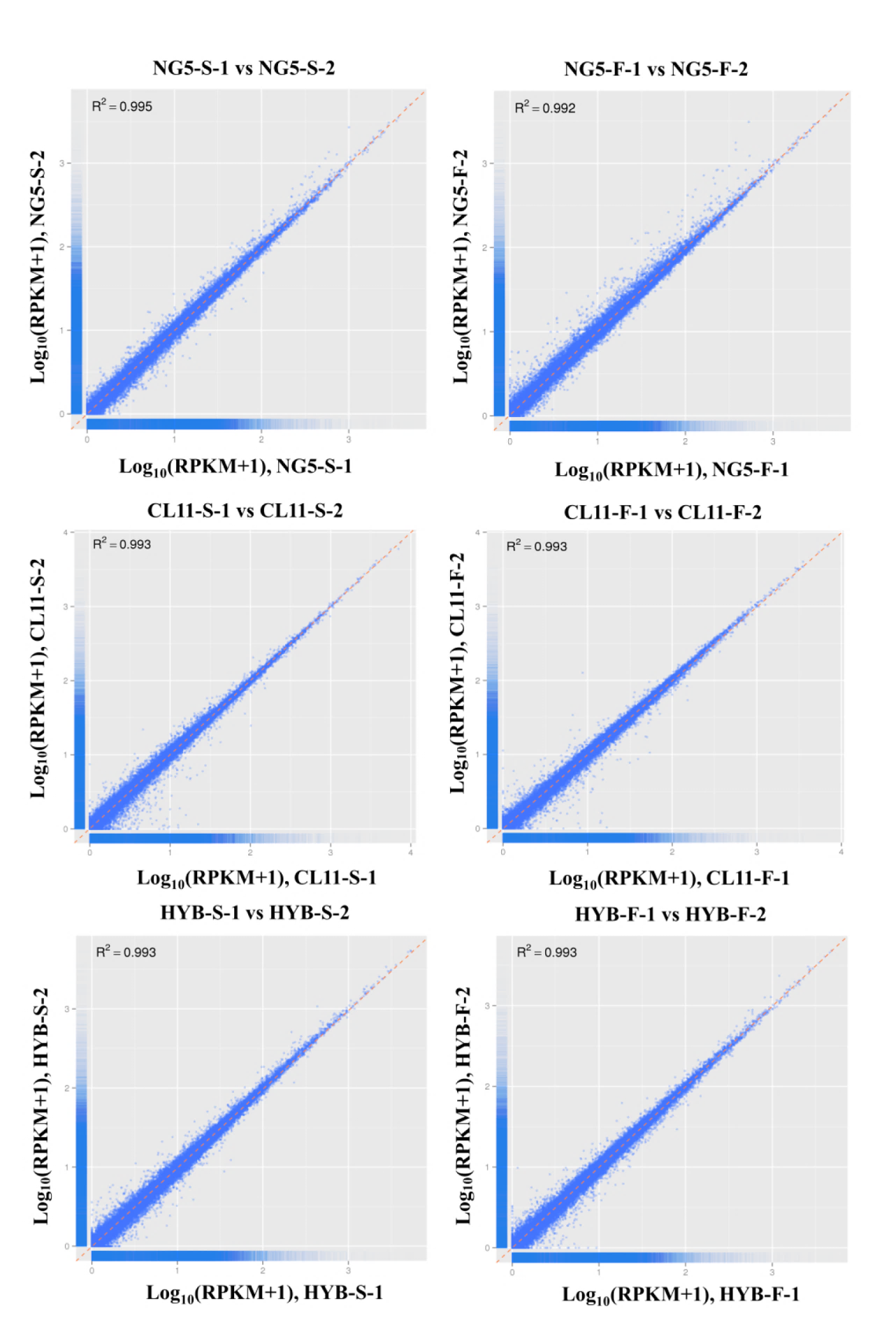
**

**Figure S1** Pearson’s correlation coefficient between two biological replicates of each genotype calculated using log_10_ (RPKM + 1). The two biological replicates were indicated by number 1 and 2. S denotes the spikelet differentiation stage; F denotes the floret differentiation stage

**
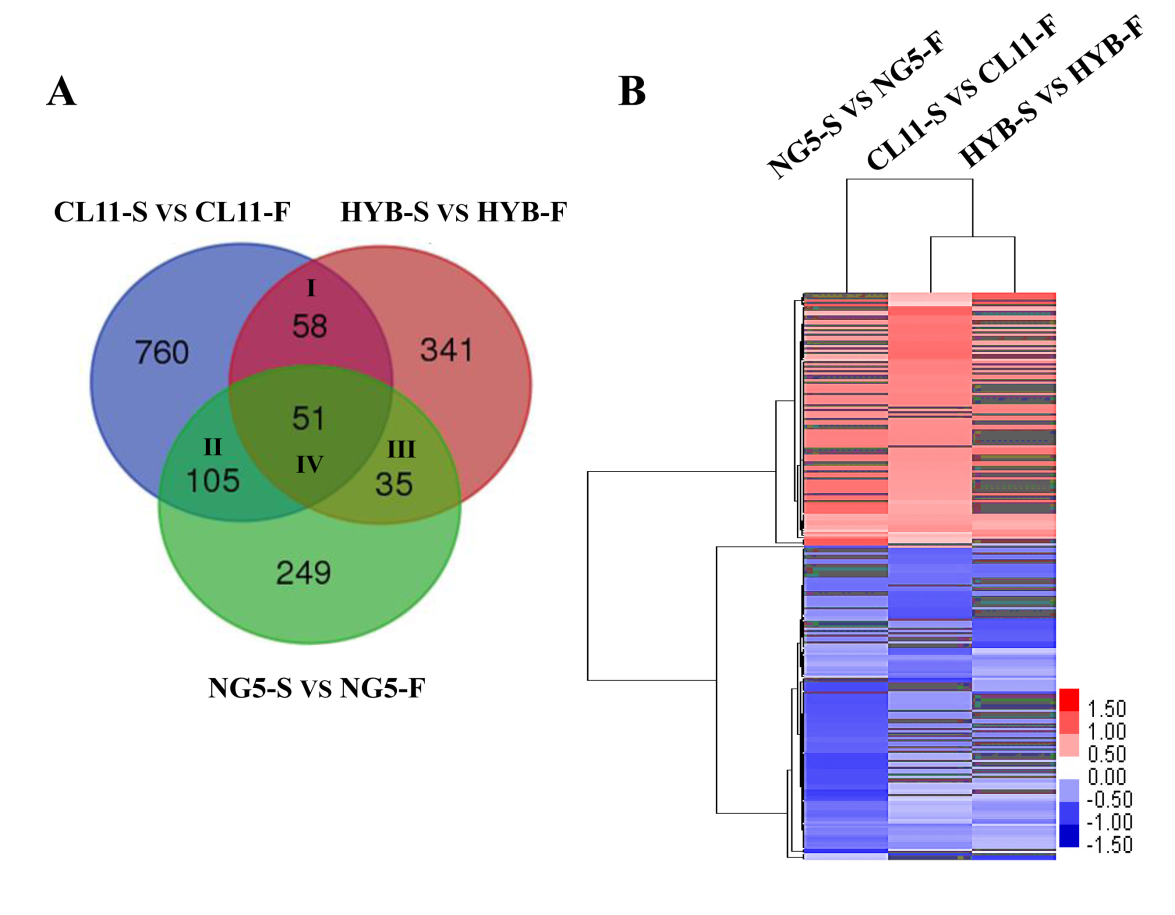
 Figure S2** Analysis of genes differentially expressed between the spikelet and floret differentiation stages in three genotypes.

(A) Venn diagram showing overlap of differentially expressed genes (DEGs) between two developmental stages in three genotypes. 249 DEGs (I+II+III+IV) were shared by at least two genotypes.

(B) Hierarchical cluster analysis of the 249 DEGs. The color key represents log_2_ (fold change). Red indicates high relative expression and blue indicates low relative expression. Gray indicates a missing value. S denotes the spikelet differentiation stage; F denotes the floret differentiation stage

**
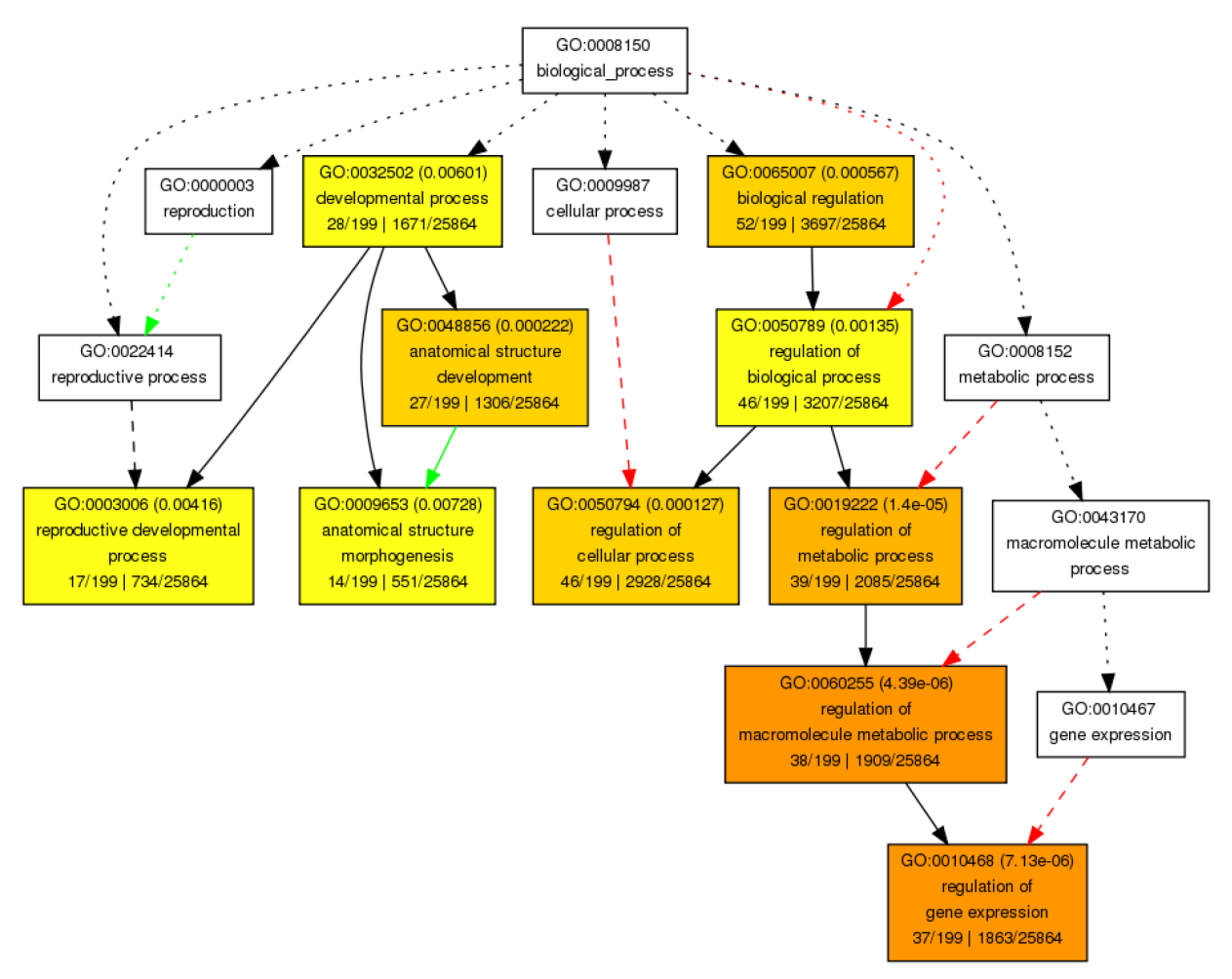
Figure S3** Directed Acyclic Graph (DAG) visualization for Gene Ontology enrichment of 249 differentially expressed genes.
