# Supplementary material for: Transcriptome profiling and comparison of maize ear heterosis during the spikelet and floret differentiation stages
Source: BMC Genomics. 2016 Nov 22;17:959. doi: 10.1186/s12864-016-3296-8 (PMC5120533; doi:10.1186/s12864-016-3296-8)
Supplement: Additional file 3: Table S2. — Distribution of gene expression levels in each genotype. (DOCX 14 kb) [file 12864_2016_3296_MOESM3_ESM.docx]

**Table S2 Distribution of gene expression levels in each genotype**

| **Genotype** | **CL11-S** | **CL11-F** | **HYB-S** | **HYB-F** | **NG5-S** | **NG5-F** | **Average** | **Percentage** |
| --- | --- | --- | --- | --- | --- | --- | --- | --- |
| **RPKM≥1** | 20,292 | 2,0423 | 20,959 | 21,258 | 20,886 | 20,891 | 20,785 | 100% |
| **20＞RPKM≥1** | 13,535 | 13,457 | 13,900 | 14,064 | 13,605 | 13,709 | 13,712 | 66.0% |
| **50＞RPKM≥20** | 3,942 | 4,133 | 4,311 | 4,468 | 4,557 | 4,471 | 4,314 | 20.8% |
| **RPKM≥50** | 2,815 | 2,833 | 2,748 | 2,726 | 2,724 | 2,711 | 2,760 | 13.3% |
