# Supplementary material for: Transcriptome profiling and comparison of maize ear heterosis during the spikelet and floret differentiation stages
Source: BMC Genomics. 2016 Nov 22;17:959. doi: 10.1186/s12864-016-3296-8 (PMC5120533; doi:10.1186/s12864-016-3296-8)
Supplement: Additional file 8: Table S5. — The selected NG5-dominant expressed genes that were assigned to different GO terms in the spikelet and floret differentiation stages. (DOCX 20 kb) [file 12864_2016_3296_MOESM8_ESM.docx]

| **Table S5** The selected NG5-dominant expressed genes that were assigned to different GO terms in the spikelet and floret differentiation stages | | | | |
| --- | --- | --- | --- | --- |
| **Spikelet differentiation stage** | |  |  |  |
| Ontology | GO term | Gene numbers | Gene ID | Annotation |
| BP | GO:0009987 | 690 | GRMZM2G001139 | MADS43 (MADS-transcription factor 43) |
|  | cellular process |  | GRMZM2G003514 | MADS-box transcription factor, *zag5*(*zea agamous5)* |
|  |  |  | GRMZM2G071620 | MADS31 (MADS-transcription factor 31) |
|  |  |  | GRMZM2G139073 | MADS-box transcription factor, *si1* (*silky1*) |
|  |  |  | GRMZM2G008691 | bhlh36 (bHLH-transcription factor 36) |
|  |  |  | GRMZM2G030123 | bHLH-transcription factor |
|  |  |  | GRMZM2G030762 | bhlh55 (bHLH-transcription factor 55) |
|  |  |  | GRMZM2G036092 | bhlh30 (bHLH-transcription factor 30) |
|  |  |  | GRMZM2G042893 | bhlh144 (bHLH-transcription factor 144) |
|  |  |  | GRMZM2G045883 | bhlh161 (bHLH-transcription factor 161) |
|  |  |  | GRMZM2G072238 | bHLH-transcription factor |
|  |  |  | GRMZM2G074438 | bhlh142 (bHLH-transcription factor 142) |
|  |  |  | GRMZM2G128807 | bhlh127 (bHLH-transcription factor 127) |
|  |  |  | GRMZM2G475289 | bhlh140 (bHLH-transcription factor 140) |
|  |  |  | GRMZM2G017187 | ARF6 (Auxin response factor 6) |
|  |  |  | GRMZM2G030710 | arftf24 (ARF-transcription factor 24) |
|  |  |  | GRMZM2G056120 | arftf11 (ARF-transcription factor 11) |
|  |  |  | GRMZM2G078274 | arftf3 (ARF-transcription factor 3) |
|  |  |  | GRMZM2G104176 | iaa7 (Aux/IAA-transcription factor 7) |
|  |  |  | GRMZM2G116557 | arftf25 (ARF-transcription factor 25) |
|  |  |  | GRMZM2G142768 | iaa18 (Aux/IAA-transcription factor 18) |
|  |  |  | GRMZM2G149481 | ARF-transcription factor |
|  |  |  | GRMZM2G378580 | arftf13 (ARF-transcription factor 13) |
|  |  |  | GRMZM2G066158 | ereb93 (AP2-EREBP-transcription factor 93) |
|  |  |  | GRMZM2G129777 | ereb192 (AP2-EREBP-transcription factor 192) |
|  |  |  | GRMZM2G132223 | ereb29 (AP2-EREBP-transcription factor 29) |
|  |  |  | GRMZM2G138396 | ereb211 (AP2-EREBP-transcription factor 211) |
|  |  |  | GRMZM2G141219 | ereb143 (AP2-EREBP-transcription factor 143) |
|  |  |  | GRMZM2G141679 | ereb109 (AP2-EREBP-transcription factor 109) |
|  |  |  | GRMZM2G368838 | ereb68 (AP2-EREBP-transcription factor 68) |
| BP | GO:0006807 | 302 | GRMZM5G872068 | gln4 (glutamine synthetase4) |
|  | nitrogen compound metabolic process |  | GRMZM2G032049 | Glutaminyl-tRNA synthetase |
|  |  |  | GRMZM2G085078 | glutamate synthase |
|  |  |  | GRMZM2G032049 | Glutaminyl-tRNA synthetase |
|  |  |  | GRMZM5G826838 | Glutamate decarboxylase |
|  |  |  | GRMZM2G330302 | umi8 (ustilago maydis induced8) |
|  |  |  | GRMZM2G170338 | Class I glutamine amidotransferase-like superfamily protein |
|  |  |  | GRMZM2G075974 | Class I glutamine amidotransferase-like superfamily protein |
|  |  |  | GRMZM2G386714 | aspartyl-tRNA synthetase |
|  |  |  | GRMZM2G136712 | aspartate kinase-homoserine dehydrogenase |
|  |  |  | GRMZM2G470556 | Probable histone acetyltransferase |
|  |  |  | GRMZM2G135970 | Aminoacylase |
|  |  |  | GRMZM2G153058 | CTP synthase family protein |
|  |  |  | GRMZM2G119583 | Acetylornithine aminotransferase |
|  |  |  | GRMZM2G700683 | carbamoyl-phosphate synthase |
|  |  |  | GRMZM2G108416 | aminotransferase domain containing protein |
|  |  |  | GRMZM2G013773 | Isoleucine--tRNA ligase |
|  |  |  | GRMZM5G887303 | Isoleucine--tRNA ligase |
|  |  |  | GRMZM2G348666 | Isoleucine--tRNA ligase |
|  |  |  | GRMZM2G082271 | Alanine--tRNA ligase |
|  |  |  | GRMZM2G029027 | Arginine--tRNA ligase |
|  |  |  | GRMZM2G148709 | Arginine--tRNA ligase |
| MF | GO:0016817 | 123 | GRMZM2G030659 | ATP-dependent RNA helicase |
|  | hydrolase activity, acting on acid anhydrides |  | GRMZM2G030873 | ATP-dependent RNA helicase |
|  |  |  | GRMZM2G031053 | Guanylate-binding protein |
|  |  |  | GRMZM2G035068 | DEAD/DEAH box RNA helicase family protein |
|  |  |  | GRMZM2G035807 | ATP-dependent RNA helicase |
|  |  |  | GRMZM2G040762 | Endoribonuclease Dicer homolog |
|  |  |  | GRMZM2G055807 | DNA repair helicase |
|  |  |  | GRMZM2G056014 | Calcium-transporting ATPase |
|  |  |  | GRMZM2G060824 | cation-transporting ATPase |
|  |  |  | GRMZM2G100732 | DNA replication ATP-dependent helicase/nuclease |
|  |  |  | GRMZM2G149520 | DEAD-box ATP-dependent RNA helicase |
|  |  |  | GRMZM2G162426 | Calcium-transporting ATPase |
|  |  |  | GRMZM2G306348 | ATP-dependent DNA helicas |
|  |  |  | GRMZM2G324462 | phospholipid-transporting ATPase |
|  |  |  | GRMZM2G326677 | Phospholipid-transporting ATPase |
|  |  |  | GRMZM2G357923 | Probable ATP-dependent RNA helicase |
|  |  |  | GRMZM2G428096 | Calcium-transporting ATPase |
|  |  |  | GRMZM2G449355 | ATP-dependent helicase |
|  |  |  | GRMZM2G469162 | DNA helicase |
|  |  |  | GRMZM2G565140 | DEAD-box ATP-dependent RNA helicase |
|  |  |  | GRMZM5G836886 | Calcium-transporting ATPase 1 |
| MF | GO:0000166 nucleotide  binding | 270 | GRMZM2G031053 | Guanylate-binding family protein |
|  |  |  | GRMZM2G031523 | Probable GTP-binding protein |
|  |  |  | GRMZM2G098129 | Interferon-induced guanylate-binding protein |
|  |  |  | GRMZM2G148709 | Arginyl-tRNA synthetase class Ic |
|  |  |  | AC155377.1_FG001 | myo1 - myosin1 |
|  |  |  | GRMZM2G171961 | ATPase putative expressed |
|  |  |  | GRMZM2G381576 | P-loop containing nucleoside triphosphate hydrolases superfamily protein |
|  |  |  | GRMZM2G111228 | GTP-binding family protein |
|  |  |  | GRMZM2G168096 | ATP binding protein putative expressed |
|  |  |  | GRMZM2G100505 | rop3 - Rho-related protein from plants 3 |
|  |  |  | GRMZM2G403149 | GTP-binding family protein |
|  |  |  | GRMZM2G423861 | kinesin-like calmodulin-binding protein |
| MF | GO:0003824 catalytic | 537 | AC203966.5_FG005 | ga20ox1(Gibberellin 20 oxidase 1) |
|  | activity |  | AC209987.4_FG002 | cytochrome P450 putative expressed |
|  |  |  | AC214350.3_FG007 | Cytochrome b-c1 complex subunit Rieske |
|  |  |  | GRMZM2G018941 | NADH-ubiquinone oxidoreductase-related |
|  |  |  | GRMZM2G024104 | Cytochrome b-c1 complex subunit Rieske,isp1 - iron-sulfur protein1 |
|  |  |  | GRMZM2G033491 | tps26 (terpene synthase26) |
|  |  |  | GRMZM2G034779 | cytochrome P450 putative expressed |
|  |  |  | GRMZM2G040209 | NADH-ubiquinone oxidoreductase |
|  |  |  | GRMZM2G069061 | Probable NADH dehydrogenase |
|  |  |  | GRMZM2G070305 | NADH dehydrogenase |
|  |  |  | GRMZM2G079348 | cat3 (catalase3) |
|  |  |  | GRMZM2G085967 | Peroxidase superfamily protein |
|  |  |  | GRMZM2G092823 | Peroxidase superfamily protein |
|  |  |  | GRMZM2G120517 | NADH-ubiquinone oxidoreductase |
|  |  |  | GRMZM2G129860 | cyp11 (cytochrome P450 11) |
|  |  |  | GRMZM2G140667 | Citrate synthase 2 |
|  |  |  | GRMZM2G320269 | APX2 (ascorbate peroxidase 2) |
|  |  |  | GRMZM2G348452 | Peroxidase 56 |
|  |  |  | GRMZM2G404249 | Cytokinin dehydrogenase |
|  |  |  | GRMZM2G088212 | cat1 (catalase1) |
