# Supplementary material for: Transcriptome profiling and comparison of maize ear heterosis during the spikelet and floret differentiation stages
Source: BMC Genomics. 2016 Nov 22;17:959. doi: 10.1186/s12864-016-3296-8 (PMC5120533; doi:10.1186/s12864-016-3296-8)
Supplement: Additional file 11: Table S6. — Comparison of the ASE patterns in hybrid between the two development stages. (DOCX 16 kb) [file 12864_2016_3296_MOESM11_ESM.docx]

**Table S6** Comparison of the ASE patterns in hybrid between the two development stages

|  |  |  | **F-stage** |  |
| --- | --- | --- | --- | --- |
| **S-stage** |  | CL11_HYB_ bias | NG5_HYB_ bias | No bias |
| CL11_HYB_ bias |  | 1,582 | 69 | 426 |
| NG5_HYB_ bias |  | 143 | 2,564 | 1,101 |
| No bias |  | 792 | 596 | 3,115 |
