# Supplementary material for: Transcriptome profiling and comparison of maize ear heterosis during the spikelet and floret differentiation stages
Source: BMC Genomics. 2016 Nov 22;17:959. doi: 10.1186/s12864-016-3296-8 (PMC5120533; doi:10.1186/s12864-016-3296-8)
Supplement: Additional file 12: Table S7. — Gene numbers of different cis- and trans-regulatory categories. (DOCX 15 kb) [file 12864_2016_3296_MOESM12_ESM.docx]

**Table S****7** **Gene numbers of different *cis*- and *trans*-regulatory variations**

| **Class** | **Classification criterion** | **S-stage** | **F-stage** |
| --- | --- | --- | --- |
| ***Cis* only** | A=B A>0 B>0 | 1,572 | 2,094 |
|  | A=B A<0 B<0 | 3,297 | 2,794 |
| Subtotal |  | 4,869 (38.5%) | 4,888 (40.8%) |
| ***Trans* only** | A>B A>0 B=0 | 103 | 144 |
|  | A<B A<0 B=0 | 1004 | 405 |
| Subtotal |  | 1,107 (8.8%) | 549 (4.6%) |
| ***Cis*-*Trans* Enhancing** | A>B A>0 B>0 | 42 | 49 |
|  | A<B A<0 B<0 | 610 | 186 |
| Subtotal |  | 652 (5.2%) | 235 (2.0%) |
| ***Cis*-*Trans* Compensating** | A>B A>0 B<0 | 44 | 45 |
|  | A>B A<0 B<0 | 335 | 211 |
|  | A<B A>0 B>0 | 225 | 195 |
|  | A<B A<0 B>0 | 214 | 120 |
|  | A>B A=0 B<0 | 161 | 151 |
|  | A<B A=0 B>0 | 237 | 238 |
| Subtotal |  | 1,216 (9.6%) | 960 (8.0%) |
| **Conserved** | A=B A=0 B=0 | 2,476 (19.6%) | 3,156 (26.3%) |
| **Ambiguous** |  | 2,317 (18.3%) | 2,205 (18.4%) |

This table shows the number and percentage of genes classified into each regulatory divergence category. A=log_2_ (CL11/NG5); B= log_2_ (CL11_HYB_/NG5_HYB_);
