# Supplementary material for: Transcriptome profiling and comparison of maize ear heterosis during the spikelet and floret differentiation stages
Source: BMC Genomics. 2016 Nov 22;17:959. doi: 10.1186/s12864-016-3296-8 (PMC5120533; doi:10.1186/s12864-016-3296-8)
Supplement: Additional file 15:Table S8. — Comparison of the cis- and trans- regulatory variations between the spikelet and floret differentiation stages. (DOCX 14 kb) [file 12864_2016_3296_MOESM15_ESM.docx]

**Table S8** **Comparison of the *cis*- and *trans-*regulatory variations between the spikelet and floret differentiation stages**

|  | ***cis* only** | ***trans* only** | ***Cis-Trans*** | **Conserved** | **Ambiguous** | **Total** |
| --- | --- | --- | --- | --- | --- | --- |
| **S-stage** | 3,931 | 980 | 1,634 | 1,928 | 1,915 | 10,388 |
| **F-stage** | 4,166 | 501 | 1,115 | 2,655 | 1,951 | 10,388 |
| **Consistent *cis*- and *trans-* regulation** | 2,575 | 174 | 434 | 1,040 | 539 | 4,762 |
| **Ratio** | 65.5% | 17.8% | 26.6% | 53.9% | 28.1% | 45.8% |
