# Supplementary material for: Transcriptome profiling and comparison of maize ear heterosis during the spikelet and floret differentiation stages
Source: BMC Genomics. 2016 Nov 22;17:959. doi: 10.1186/s12864-016-3296-8 (PMC5120533; doi:10.1186/s12864-016-3296-8)
Supplement: Additional file 16: Table S9. — qRT-PCR validation of differentially expressed genes in the spikelet and floret differentiation stages from RNA-seq. (DOCX 20 kb) [file 12864_2016_3296_MOESM16_ESM.docx]

**Table S9 qRT-PCR validation of differentially expressed genes in the spikelet and floret differentiation stages from RNA-seq**

|  | **geneID** | | **RNA-seq**  **(HYB-S vs. NG5-S)** | **QRT-PCR**  **(HYB-S vs. NG5-S)** | **Stdev** | **Forward primer** | | **Reverse primer** | |  |  |
| --- | --- | --- | --- | --- | --- | --- | --- | --- | --- | --- | --- |
| **S-stage** | GRMZM2G158394 | | 2.8707 | 2.893354098 | 0.35 | GCGACTTCTACATAACGGGCTT | | GCTGGGGCACCTGATGTTG | |  |  |
|  | GRMZM2G112830 | | 0.67528 | 0.953493754 | 0.11 | CTCGCCTTCGGACAGGATAA | | GCCCTGTGCGTTGGCTACTA | |  |  |
|  | GRMZM2G172214 | | -0.4767 | -0.651434898 | 0.36 | GCTCCCTCCTCAGCTACGAC | | GTGGACATGGTCTCGCTCAG | |  |  |
|  | GRMZM2G700208 | | 0.94449 | 1.690388044 | 0.17 | GCTCCCTCCTCAGCTACGAC | | GTGGACATGGTCTCGCTCAG | |  |  |
|  | GRMZM2G146308 | | 0.18006 | 1.471170584 | 0.51 | CTCAGTCCTTCTCGGCTTCCT | | GCACCAAACCACTTCTCATCCT | |  |  |
|  | GRMZM2G376416 | | 0.87635 | 1.39723587 | 0.39 | GCAAATTGCTGGAACACCTG | | CCCTGAACTCGTCGTCGTAC | |  |  |
|  | GRMZM2G038677 | | 0.53038 | 0.535791079 | 0.53 | CGTCACGTCCTACGACTGGG | | GCGAACCTGTGCATGTTGC | |  |  |
|  | GRMZM2G150683 | | -0.63637 | -0.169644038 | 0.06 | GCGAACCTGTGCATGTTGC | | ACAGGTTTTCTTCGGGTCTTC | |  |  |
|  | GRMZM2G113513 | | -0.62515 | -0.106046359 | 0.05 | GGGATAAGGTGACCGACGTAC | | GTCCTGTCTGGCCTTTGCA | |  |  |
|  |  | **geneID** | **RNA-seq**  **(HYB-F vs. CL11-F)** | **QRT-PCR**  **(HYB-F vs. CL11-F)** | **Stdev** | | **Forward primer** | | **Reverse primer** | |  |
| **F-stage** | GRMZM2G152417 | | -0.87569 | -0.156400839 | 0.02 | ACACCTGGGCTGACACCTATC | | GCTCCTGACATTGGGACACC | |  |  |
|  | GRMZM2G114924 | | 2.5379 | 2.751231511 | 0.09 | AGACGGACCAGCAGAAGAGG | | GGGGCAGTGATTTCACCAAA | |  |  |
|  | GRMZM2G125529 | | 3.3893 | 3.286472638 | 0.34 | GTAAATATGTAGGAAATCGGCCA | | ATACTGACAGACGATGAACGAG | |  |  |
|  | GRMZM2G009091 | | 0.68145 | 1.252871513 | 0.02 | GTCACCTGTTGATGGGAAGCC | | TCCAACGAATGGTGCGATACA | |  |  |
|  | GRMZM2G128228 | | 0.62132 | 0.917489815 | 0.18 | CGAGTCATACCCCAGATTTTAGA | | TGTTGTGGCTACTTCGTTCTTG | |  |  |
|  | GRMZM2G170101 | | -0.60595 | -0.6060613 | 0.10 | TTCAGGGACGCCGTGGA | | GGTATGAACTTTGCTCCTATGGC | |  |  |
|  | GRMZM2G105401 | | 3.7053 | 2.064449628 | 0.40 | GCAACAGTTTTGAAGTCCACGA | | TACTTTATTCCTCCGCAGGGT | |  | |
|  | GRMZM2G022107 | | 0.74009 | 0.754713694 | 0.06 | AGGTTGCTGCTTCCATTGTG | | TCCCTCGTTCCAGTGCTTG | |  |  |
