# Supplementary material for: Transcriptome profiling and comparison of maize ear heterosis during the spikelet and floret differentiation stages
Source: BMC Genomics. 2016 Nov 22;17:959. doi: 10.1186/s12864-016-3296-8 (PMC5120533; doi:10.1186/s12864-016-3296-8)
Supplement: Additional file 17: Table S10. — Verified SNPs locus in ASE analysis. (DOCX 37 kb) [file 12864_2016_3296_MOESM17_ESM.docx]

**Table S10** **Verified SNPs locus in the ASE analysis**

| **Chr.** | **gene ID** | **Position** | **ref** | **NG5** | **CL11** | **Primers** |  |
| --- | --- | --- | --- | --- | --- | --- | --- |
| **1** | GRMZM5G862565 | 100356569 | A | T | A | AGAAACTGCTGGCACAAGACAA |  |
|  |  | 100356697 | G | G | A | GGAAAGTAACAATAAAGCGGAGG |  |
| **1** | GRMZM2G086179 | 10434646 | C | A | C | CTGCTGATGCCTTCACCACA |  |
|  |  | 10434653 | A | C | A | GAACTCCGATCCAAGACCAATT |  |
|  |  | 10434904 | C | A | C |  |  |
| **1** | GRMZM2G012306 | 106904750 | C | C | G | CCATCAACCTGGGCATCTTC |  |
|  |  | 106904819 | C | C | G | CCTCCACGGACACCAACGA |  |
| **2** | GRMZM2G360907 | 102286405 | C | A | C | GGGGAACAATCTTTCACAGTCA |  |
|  |  | 102286450 | C | T | C | GGTAAGCAGACGGCAAGTAATC |  |
| **2** | GRMZM2G089010 | 104593496 | T | T | C | CATGAATCCAGAGCAAAGGG |  |
|  |  | 104593670 | C | C | T | GTAGGGTAACAGAACCACCACAA |  |
|  |  | 104593685 | T | T | C |  |  |
| **2** | GRMZM2G007791 | 9974893 | G | G | A | CTCACGCCATCACGGAAGTA |  |
|  |  | 9974989 | G | G | A | GCAAACAATCGAAAGGGACA |  |
| **2** | GRMZM2G007914 | 9972702 | G | A | G | ACCCTGTCAAAAGCAGAAGAGAT |  |
|  |  | 9972730 | G | C | G | AGACCAACAAAATGTCCGATGA |  |
| **3** | GRMZM2G028637 | 10095964 | A | A | G | TGGCATTCCCTTTCTTCTTTG |  |
|  |  | 10096048 | C | C | A | GCTGCTATCATCGTCTCCGT |  |
| **3** | GRMZM2G352159 | 107591821 | C | G | C | CTGTCTGCCTCACCTGCTCG |  |
|  |  | 107591852 | A | C | A | TATCTTACCCTGCCCCTTGC |  |
| **3** | GRMZM2G059703 | 114801803 | G | A | G | CATTGCCATTTAATTGAGGGA |  |
|  |  | 114801882 | C | A | G | CAGAAGCCATTCCGAAGTGAT |  |
| **3** | GRMZM2G457411 | 115025072 | C | A | C | TTTAGCCTATTAGTTGCGGTATTG |  |
|  |  | 115025127 | G | G | A | ACTGTCGCTTCTGCTTCCTG |  |
| **3** | GRMZM2G320452 | 115811324 | C | T | C | GCAGTCGCTCCTGTTGTCCA |  |
|  |  | 115811349 | G | C | G | GCCATGATCCGCTCCTTACC |  |
| **4** | GRMZM2G068331 | 10235947 | G | G | A | AGCAACGCACTGTCCATTTATC |  |
|  |  | 10235986 | A | A | G | AGAACATTCCGTGGCATCTATC |  |
| **4** | GRMZM2G021225 | 114353426 | A | A | C | TTCAGCACAGACATCACCAGAG |  |
|  |  | 114353442 | G | A | G | GAGGGGACACCATTGACAGC |  |
|  |  | 114353496 | C | C | T |  |  |
| **4** | GRMZM2G133675 | 6605771 | T | T | C | TCACCACTTGCCTTGTTCAGA |  |
|  |  | 6605804 | C | T | C | CTGTCAGTTGGCTACCCGTC |  |
|  |  | 6605860 | G | G | A |  |  |
|  |  | 6605928 | C | C | A |  |  |
| **4** | GRMZM2G011364 | 5707950 | C | T | C | CAAACCGAACCTTTCACAGC |  |
|  |  | 5707967 | A | G | A | AATCAGCACGGGACACTCAA |  |
|  |  | 5707975 | T | C | T |  |  |
| **5** | GRMZM2G079653 | 100107651 | T | T | C | ACAACAGGTTAGTAGCACAAGCAC |  |
|  |  | 100107670 | T | T | G | GGTTTTACGCCGATGAGGAG |  |
|  |  | 100107756 | A | A | G |  |  |
| **5** | GRMZM2G181028 | 10113431 | A | G | A | CGAGGAAGACGGAAATAAATACA |  |
|  |  | 10113497 | C | T | C | GGATGGTGCTCTGAGTGGAAT |  |
| **5** | GRMZM2G152548 | 10241039 | T | T | C | AACGGCAATCTGTAAAACGAG |  |
|  |  | 10241119 | G | G | A | CAACGGTCAGGTCAAAGGAG |  |
|  |  | 10241214 | C | C | T |  |  |
| **5** | GRMZM2G158359 | 10428672 | G | G | A | CTGGACGAGTGACCACAAAAG |  |
|  |  | 10428678 | G | G | T | CAGCATACACGACCAAAGAGC |  |
|  |  | 10428684 | G | C | G |  |  |
| **6** | GRMZM5G856297 | 151084262 | T | T | C | CACCAAGCACAGCACCCCTAC |  |
|  |  | 151084297 | C | C | T | TGGAACTGTTGATGCGAGCC |  |
| **7** | GRMZM2G006752 | 133193321 | A | G | A | TGGTAGTGATTGTTTCGGAGGTA |  |
|  |  | 133193410 | C | T | C | CCTGATTTGGCTGGGAGTTCTA |  |
|  |  | 133193499 | C | T | C |  |  |
|  |  | 133193574 | A | T | A |  |  |
| **7** | GRMZM2G087144 | 141845309 | A | T | A | AACACCAGAACGCTTCATCG |  |
|  |  | 141845384 | G | C | G | GAATGGCTACAAGGGAAATGG |  |
|  |  | 141845473 | A | A | T |  |  |
| **7** | GRMZM2G141517 | 4083323 | A | T | A | GGCCAAGTAGTAACAGACAGCA |  |
|  |  | 4083413 | A | A | G | CTTCAATGCCACATCCTTCG |  |
|  |  | 4083776 | G | A | G |  |  |
|  |  | 4083794 | G | C | G |  |  |
|  |  | 4083863 | A | G | A |  |  |
|  |  | 4083893 | T | C | T |  |  |
| **8** | GRMZM2G110063 | 14779811 | A | G | A | GAGGAGCCAAGCCTGTAGTGA |  |
|  |  | 14779859 | C | C | T | GAGGAAACGAACAGCAAGGAA |  |
|  |  | 14780138 | A | G | A |  |  |
| **8** | GRMZM2G060045 | 134275940 | T | C | T | TTCTGGCTGAAACCATCCCC |  |
|  |  | 134275996 | G | A | G | ATCAACCGTTCCTCCCCTGT |  |
| **8** | GRMZM2G035933 | 168485752 | A | G | A | ACCTCACTACCTGCTCCTCACTCC |  |
|  |  | 168485909 | T | G | T | CCTCAAACTCGCTCGCTACACC |  |
|  |  | 168485929 | T | T | C |  |  |
|  |  | 168485974 | C | C | T |  |  |
|  |  | 168486085 | G | C | G |  |  |
|  |  | 168486134 | G | A | G |  |  |
|  |  | 168486217 | T | T | C |  |  |
| **8** | GRMZM5G840487 | 169071791 | A | A | G | CAACTACACTCCTCTGAACTCTCCA |  |
|  |  | 169071924 | T | T | C | CGAACAGACTCGCCTTGAGGA |  |
|  |  | 169071932 | T | T | C |  |  |
|  |  | 169071993 | A | A | T |  |  |
|  |  | 169072177 | A | A | G |  |  |
|  |  | 169072183 | G | G | A |  |  |
|  |  | 169072274 | T | T | C |  |  |
| **9** | GRMZM2G403151 | 19238582 | C | T | C | AAAACAACAAACCAACTCCAAATC |  |
|  |  | 19238691 | G | A | G | TGCCGTCTTCTGAGGTCACTAC |  |
| **9** | GRMZM2G006721 | 148058199 | T | C | T | CCATTTCATTTCTCTACCTCTCCAG |  |
|  |  | 148058211 | A | G | A | TTACATTGACTCGCCGTTGC |  |
|  |  | 148058354 | T | C | T |  |  |
|  |  | 148058420 | A | C | A |  |  |
|  |  | 148058438 | G | A | G |  |  |
| **9** | GRMZM2G017831 | 148931908 | T | T | C | CATGTCAGCTTGAAGACAAACGC |  |
|  |  | 148931998 | A | G | A | TGATCCGACCATAGTTGCAAATT |  |
|  |  | 148932295 | A | A | G |  |  |
|  |  | 148932316 | G | G | A |  |  |
|  |  | 148932399 | C | C | T |  |  |
|  |  | 148932592 | T | C | T |  |  |
| **10** | GRMZM2G140919 | 140509709 | A | A | G | AGAGGAGTTGGATCACGGAGC |  |
|  |  | 140509723 | G | G | A | ATGGTGCGGAAGAACAGAGC |  |
|  |  | 140509788 | G | G | A |  |  |
| **10** | GRMZM2G004414 | 4685185 | G | C | G | CATTGAACCAGACAGACAGATTG |  |
|  |  | 4685239 | T | G | T | TCTATGGTCCGTCGTGCTCTT |  |
|  |  | 4685425 | G | C | G |  |  |
|  |  | 4685589 | G | A | G |  |  |
|  |  | 4685674 | T | A | T |  |  |
| **10** | GRMZM2G112782 | 71064913 | T | T | A | CATATCTTAATGGGCGTTGTTC |  |
|  |  | 71065095 | T | T | A | GGATCTGGCTCAGAGGAGGT |  |
|  |  | 71065155 | G | G | A |  |  |
|  |  | 71065369 | T | A | T |  |  |
|  |  | 71065381 | A | G | A |  |  |
| **10** | GRMZM2G372475 | 80732224 | G | G | C | ACGGTGAGGCTGAGCAACA |  |
|  |  | 80732381 | G | A | G | CTCGGTTTGACTCTCAATGAGAAAG |  |
|  |  | 80732391 | C | C | G |  |  |
|  |  | 80732610 | C | C | T |  |  |
|  |  | 80732685 | G | G | A |  |  |
